# Phylogenetic relationships and taxonomic position of genus *Hyperacrius* (Rodentia: Arvicolinae) from Kashmir based on evidences from analysis of mitochondrial genome and study of skull morphology

**DOI:** 10.7717/peerj.10364

**Published:** 2020-11-18

**Authors:** Natalia I. Abramson, Fedor N. Golenishchev, Semen Yu. Bodrov, Olga V. Bondareva, Evgeny A. Genelt-Yanovskiy, Tatyana V. Petrova

**Affiliations:** 1 Department of Molecular Systematics, Zoological Institute Russian Academy of Sciences, Saint-Petersburg, Russian Federation; 2 Department of Mammals, Zoological Institute Russian Academy of Sciences, Saint-Petersburg, Russian Federation

**Keywords:** Mitogenome, Phylogeny, Taxonomy, Rodents, Arvicolinae, Hyperacrius, Museum DNA

## Abstract

In this article, we present the nearly complete mitochondrial genome of the Subalpine Kashmir vole *Hyperacrius fertilis* (Arvicolinae, Cricetidae, Rodentia), assembled using data from Illumina next-generation sequencing (NGS) of the DNA from a century-old museum specimen. De novo assembly consisted of 16,341 bp and included all mitogenome protein-coding genes as well as 12S and 16S RNAs, tRNAs and D-loop. Using the alignment of protein-coding genes of 14 previously published Arvicolini tribe mitogenomes, seven Clethrionomyini mitogenomes, and also *Ondatra* and *Dicrostonyx* outgroups, we conducted phylogenetic reconstructions based on a dataset of 13 protein-coding genes (PCGs) under maximum likelihood and Bayesian inference. Phylogenetic analyses robustly supported the phylogenetic position of this species within the tribe Arvicolini. Among the Arvicolini, *Hyperacrius* represents one of the early-diverged lineages. This result of phylogenetic analysis altered the conventional view on phylogenetic relatedness between *Hyperacrius* and *Alticola* and prompted the revision of morphological characters underlying the former assumption. Morphological analysis performed here confirmed molecular data and provided additional evidence for taxonomic replacement of the genus *Hyperacrius* from the tribe Clethrionomyini to the tribe Arvicolini.

## Introduction

Genus *Hyperacrius* Miller, 1896 is among the understudied taxonomic groups within the subfamily Arvicolinae Gray, 1821 (Rodentia: Cricetidae). The range of *Hyperacrius* is restricted to mountainous areas of Kashmir and western Pakistan, particularly moist temperate forest and grassy slopes located between 1,850 and 3,050 m of altitude (Agrawal, 2000; Pardiñas et al., 2017). While Hy*peracrius* is often mentioned in phylogenetic reconstructions of Arvicolinae, most of the recent papers (Carleton & Musser, 2005; Robovský, Řičánková & Zrzavý, 2008; Kohli et al., 2014) usually refer to a comprehensive morphological description dating back to the mid-20th century (Phillips, 1969).

The subgenus *Hyperacrius*, with the type species *Arvicolа fertilis* True, 1894 was firstly described by Miller (1896). Further, Hinton (1926) elevated the rank of the *Hyperacrius* to the level of genus, assuming that this taxon represents an ancient and highly specialized branch that can be placed close to the genus *Alticola* Blanford, 1881. Following this assumption, Hooper & Hart (1962) and later Gromov & Polyakov (1977) considered *Hyperacrius* as a member of tribe Clethrionomyini Hooper et Hart, 1962 (= Myodini Kretzoi, 1955). In this article, we henceforward use the genus name *Clethrionomys* Tilesius, 1850 and Clethrionomyini for the tribe instead of *Myodes* Pallas, 1811 and Myodini. The detailed arguments for this decision are given in Tesakov et al. (2010) and Krystufek et al. (2020).

Currently, the genus includes two species, *H. fertilis* (True’s vole*)* and *H. wynnei* Blanford, 1881 (Murree vole). Taxonomic status of the genus *Hyperacrius* was substantiated by a combination of morphological features, including cementless and rootless molars, characteristics of the upper and lower molar patterns, and the structure of hard palate (Miller, 1896; Hinton, 1926; Gromov & Polyakov, 1977). Later, *Hyperacrius* and *Alticola* were united in a new subtribe Alticoli of the tribe Clethrionomyini based on the analysis of skull morphology (Gromov & Polyakov, 1977). This phylogenetic affinity with *Alticola* was later confirmed by several authors (Corbet, 1978; Corbet & Hill, 1992; Carleton & Musser, 2005). Due to the lack of sampling in the wild, only a few attempts to review the taxonomic status of *Hyperacrius* have been implemented.

The first genetic dataset on *Hyperacrius* included sequencing of the fragments of mitochondrial cytochrome *b* gene (810 bp) and the *G6pd* gene intron (255 bp) (NCBI accession numbers KJ556725 and KJ556610 respectively) from a museum specimen (Kohli et al., 2014). The authors used these sequences of *Hyperacrius* for the reconstruction of phylogenetic relationships within the tribe Clethrionomyini; their taxonomic dataset consisted of all the species from this tribe and only a few genera from other tribes in the subfamily Arvicolinae as an outgroup. Thus, although this work was the first where the position of *Hyperacrius* within Clethrionomyini was doubted (sister to Clethrionomyini according to cytochrome *b* phylogenetic reconstruction and within a clade of *Microtus* spp. according to G6pd), the data obtained were still insufficient to clarify the genus phylogeny and taxonomic position within the subfamily.

In this study, we implemented shotgun sequencing to obtain the mitochondrial genome of *Hyperacrius fertilis* from a century-old museum specimen. Combining our data with already published mitogenome sequences of other Arvicolinae, and reviewing the taxonomically significant morphological characteristics of the *H. fertilis* specimen, we were aimed to revise the phylogenetic relationships and taxonomic position of *Hyperacrius* genus within the Arvicolinae subfamily.

## Materials and Methods

### Material

The voucher specimen studied is stored in the collection of Zoological Institute RAS and labeled as *Hyperacrius fertilis brachelix* No. 29256, coll. No. 76; collected 3.09.1903; skin and skull (without neurocranium and right lower jaw), male, Kashmir, 2,133.6 m altitude. The specimen was obtained as a donation from the Natural History Museum (London, UK) in 1937. The morphological diagnostic traits of the specimen were verified in accordance with descriptions in Phillips (1969) and Gromov & Polyakov (1977). For the phylogenetic reconstructions, annotated complete mitochondrial genome sequences of 23 species of Arvicolinae were mined from NCBI GenBank Nucleotide database (see Table S1 for details). Skulls of *Alticola argentatus* and *Microtus socialis* were taken from the collection of Zoological Institute (collection ZIN IDs 74245 and 77664, respectively).

### DNA extraction, library preparation and sequencing

To reduce the potential contamination, all manipulations with the museum specimen were carried out in a separate laboratory room isolated from post-PCR facilities, predominantly being used for studies of historic samples from the collection of Zoological Institute. All the working surfaces, instruments and plastics were sterilized with UV light and chloramine-T. DNA from the museum skin sample (2 × 2 mm piece from the inner side of the lip, dissected by a sterilized surgical blade) was isolated using the phenol-chloroform extraction method according to a standard protocol (Köchl, Niederstätter & Parson, 2005). PCR was prepared using a PCR workstation (LAMSYSTEMS CC, Miass, Russia). As the studied specimen was 117 years old, several steps were implemented to ensure that the obtained sequence is free of deamination sequencing errors. Thereby, DNA library preparation was implemented using the NEBNext Ultra II DNA Library Prep Kit for Illumina (New England Biolabs, Ipswich, MA, USA), which is characterized by containing the uracil-DNA glycosylase in the Prep Enzyme Mix, the component used at the first, NEBNext End Prep of the protocol for library preparation. This monofunctional DNA glycosylase catalyzes the hydrolysis of the *N*-glycosidic bond from deoxyuridine to release uracil and thus reduces the amount of hydrolytic deamination.

DNA quality was checked with Qubit, final library length distribution and checking for the absence of adapters was performed using Bioanalyzer 2100 (Agilent, Santa Clara, CA, USA). Sequencing was performed on Illumina HiSeq 4000 system with a 75 bp read length at the Skoltech Genomics Core Facility (https://www.skoltech.ru/research/en/shared-resources/gcf-2/).

### Mitogenome de-novo assembly, annotation and sequence analyses

The quality of raw reads was evaluated using FastQC (Andrews, 2010). Raw reads were assembled to a reference complete mitochondrial genome of *Clethrionomys glareolus* (NCBI accession KM892835) using Geneious Prime 2019.1 (Biomatters Ltd., Auckland, New Zealand). We then subsequently used mapDamage 2.0 (Jónsson et al., 2013) to display nucleotide misincorporation patterns that can often be observed during the studies of ancient DNA isolated from the museum samples as a result of severe post-mortem DNA damage. De novo assembly was implemented using plasmidSPAdes (Bankevich et al., 2012) with the default settings. The resulting contigs were filtered by length, and contigs with the most similarity in size to mitochondrial DNA were selected (considering mammal mitogenome length of ca. 16 kb). All positions of low quality, low coverage, as well as fragments that greatly differed from the reference Arvicolinae mitochondrial genomes, were replaced by N manually. Assembled sequences of protein-coding genes were checked for internal stops in PCGs manually.

The contigs were annotated using the MITOS web server (Bernt et al., 2013), with the default settings. Gene boundaries were checked and refined by alignment against 21 published mitogenome sequences of Arvicolinae (see details in Table S1). Mitochondrial genomes were aligned with Mauve 1.1.1 (Darling et al., 2004) implemented as a plugin for Geneious Prime 2019.1. Concatenated nucleotide alignment of 13 protein-coding genes was firstly performed using MAFFT version 7.222 (Katoh et al., 2002), and subsequently translated based on vertebrate mtDNA genetic code. Codon usage was calculated using Geneious Prime 2019.1. GC-skew was analyzed with the BioSeqUtils package in BioPython (Cock et al., 2009) under the Python 3.0 environment. Nucleotide composition was evaluated using Mega v. 10.1.7 (Kumar et al., 2018).

### Phylogenetic analyses

Phylogenetic reconstructions were conducted from the alignment of 13 protein-coding genes consisting de novo assembled mitogenome of *H. fertilis* and 24F mitochondrial genomes of representatives of Arvicolini and Clethrionomyini tribes, *Dicrostonyx torquatus* and *Ondatra zibethicus*, as an outgroup. The final alignment also included the only published fragment of mitochondrial *cytochrome b* sequence of *H. fertilis* (KJ556725) to prove the authenticity of de-novo assembled *H. fertilis* mitogenome and two *CYTB* sequences of genus *Alticola* (*A.stoliczkanus* and *A.tuvinicus*). Prior to performing the phylogenetic reconstructions, a disparity index test (Kumar & Gadagkar, 2001) measuring the homogeneity of substitution patterns between sequences was calculated in MEGA 10.1.7 (Kumar et al., 2018). Since third codon position has previously been shown to bias phylogenetic reconstructions (Breinholt & Kawahara, 2013), additional alignment of protein-coding genes, where transitions in 3rd codon position were masked by RY-coding (R for purines and Y for pyrimidines), was developed. Thus, three datasets were subsequently analyzed—total alignment of 13 protein-coding genes, where all three codon positions were considered (with a length of 11,391 bp), RY-coded alignment with transitions in third codon position masked, and 7,594 bp alignment where 3rd codon position was removed.

To select the optimal partitioning scheme, we used PartitionFinder 2.1.1 (Lanfear et al., 2017) applying AICc and “greedy” algorithm, when an analysis is based on the a priori features of the alignment. Our analysis started with the partitioning by codon positions within protein-coding gene fragments, each treated as a unique partition, and optimal subsets were finally chosen by PartitionFinder (Table S2).

Maximum Likelihood (ML) analysis was performed using IQ-TREE web server (Trifinopoulos et al., 2016) with 10,000 ultrafast bootstrap replicates (Hoang et al., 2018). Bayesian Inference (BI) analysis was performed in MrBayes 3.2.6 (Ronquist et al., 2012). Each analysis started with random trees and performed two independent runs with four independent Markov Chain Monte Carlo (MCMC) for 5 million generations with sampling every 1,000th generation, the standard deviations of split frequencies were below 0.01; potential scale reduction factors were equal to 1.0; stationarity was examined in Tracer v1.7 (Rambaut et al., 2018). A consensus tree was constructed based on the trees sampled after the 25% burn-in. Phylogenetic reconstructions using ML and BI analyses were performed on three datasets uniformly.

In order to check whether the resulting sequence is chimeric, we conducted BI analysis for each PCG separately (with partitions by codon positions), models yielded from the PartitionFinder, and the other BI run parameters were similar to those used in the analysis of the complete PCG dataset. The *cytochrome b* tree was reconstructed from the combined dataset of taxa from this study and data from of Kohli et al. (2014) that analyzed multilocus systematics of Clethrionomyini including the first published sequence of *H. fertilis*.

### Morphological analysis

Skull measurements were taken using a digital caliper «Mitutoyo-15» and included the following: length of diastema; zygomatic breadth; interorbital breadth; length of incisive foramina; length of nasals; length of lower jaw; length of upper molar row; length of lower molar row; length of upper molar (M3); length of lower molar (m1). The digital pictures of each skull were taken using stereomicroscope equipped with Canon EOS 60D camera and further processed in Helicon Focus proprietary software. To obtain drawings of the teeth, a Zeiss Stemi SR binocular equipped with a camera lucida was used.

## Results

### Raw reads analysis

The study of museum specimens requires a lot of additional quality checks of raw reads. First of all, deamination problems that occur with long-term DNA storage as an excess of cytosine to thymine (C-to-T) misincorporations at 5′ ends of sequences and complementary guanine to adenine (G-to-A) misincorporations at 3′ ends, due to enhanced cytosine deamination in single-stranded 5′-overhanging ends. The mapDamage analysis showed a low variation of deamination misincorporations values (Fig. S1). C to T misincorporations (red) varied from 12.09% to 17.94%, G to A (blue) form 12.84–17.49%. Levels of misincorporations compared with all other substitution variants, colored in gray and also similar to values from papers (Molto et al., 2017).

### Mitochondrial genome composition

**Figure 1:**
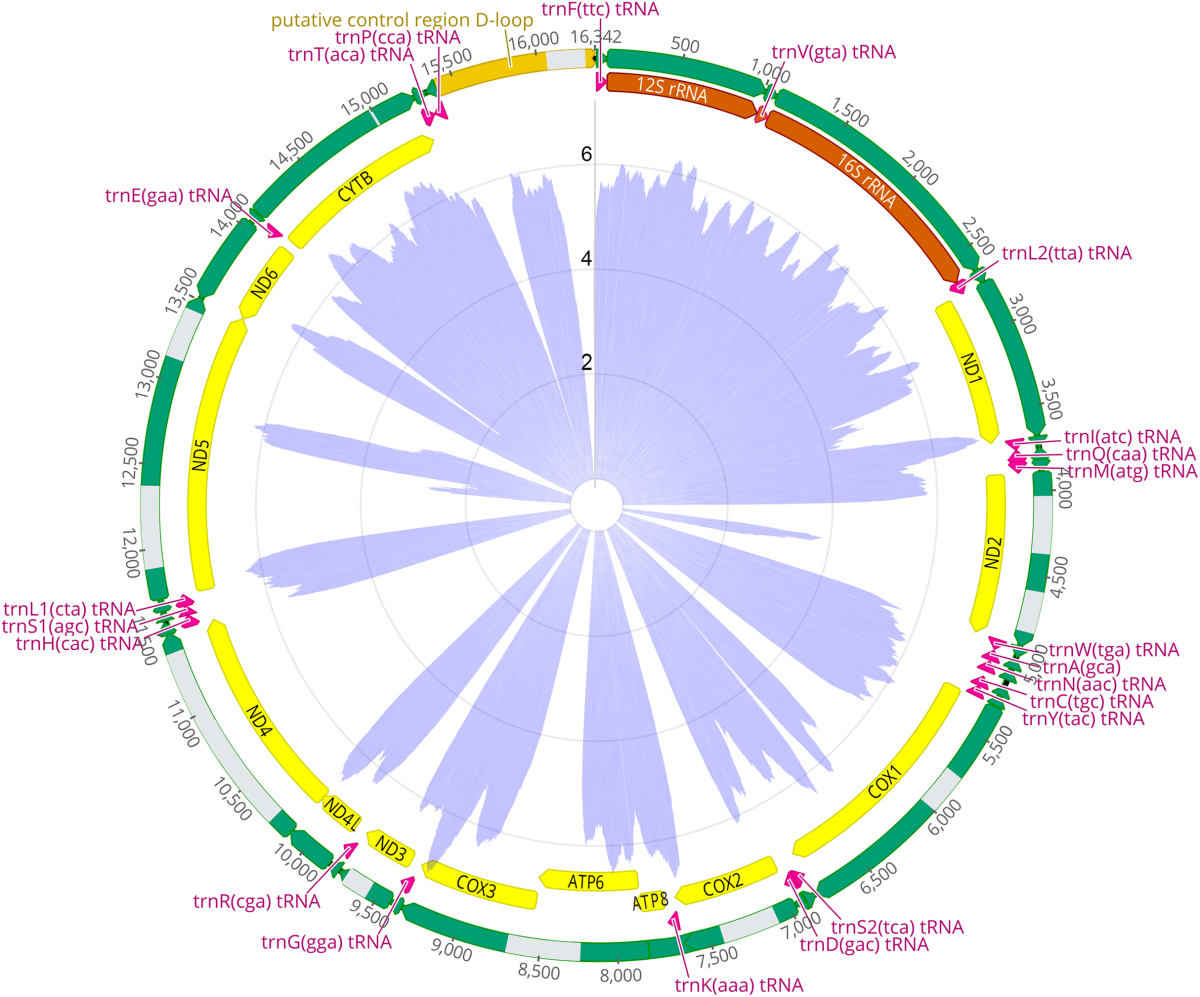
Map of the mitochondrial genome of *Hyperacrius fertilis* mapped onto the mitochondrial genome of *Clethrionomys glareolus* (NCBI accession KM892835). Yellow pointed bands mark annotations of protein-coding genes (CDs); rRNAs are marked ****in vermilion, tRNAs in violet. Sequenced areas are shown in green, non-sequenced areas are marked in gray. Log-transformed values of coverage of the NGS sequencing, that is, the number of unique reads that include a given nucleotide in the reconstructed sequence of *H. fertilis* against the reference of *C. glareolus* is indicated by a circular barplot in blue in the center of the figure. The numbers indicate the logarithmic values of the coverage

The mitogenome of *Hyperacrius fertilis* is a closed-circular molecule of 16,341 bp in length (GenBank accession No. MT433094, Fig. 1). In spite of full-length alignment of all reads to the *Clethrionomys glareolus* complete mitochondrial genome 25% of reads consensus was masked by N because of low quality and coverage (Fig. S2). The mitochondrial genome contains the typical set of 13 PCGs, two ribosomal RNA genes (rrnL and rrnS), 21 transfer RNA genes (tRNAs), and a putative control region (Fig. 1; Table S3). Nine genes (ND6 and eight tRNAs) were oriented in the reverse direction, whereas the others were transcribed in the forward direction. Unfortunately, several of the PCGs *(COX1, COX2, ATP6, COX3, ND2, ND3, ND4, ND5)* were represented by partial sequences (Fig. 1; Table 1). The gene order and organization of *H. fertilis* are consistent with other mitochondrial sequences of rodents. The mitogenome of *H. fertilis* harbors a total of 53 bp overlapping sequences in four regions. The longest overlap of 42 bp in length is located between *ATP8* and *ATP6*. All tRNAs have the typical cloverleaf structure, similar to those reported in most animal mitogenomes. The nucleotide composition is significantly biased (A, C, G, and T was 29.1%, 26.7%, 14.6%, and 29.6%, respectively) with G + C contents of 27.4%. The GC-skew is defined by GC-skew = (G − C)/(G + C) and constitutes −0.20.

**Table 1:** List of the 13 protein-coding genes in the mitochondrial genome of *Hyperacrius fertilis*.

Gene	Start	Stop	Length	Direction	fcd	scd	Completeness	Absent fragments, aa
*CYTB*	14,112	15,254	1,143	forward	ATG	TAA	complete	
*ND6*	13,513	14,037	525	reverse	ATG	TAA	complete	
*ND5*	11,705	13,516	1,812	forward	ATA	TAA	partial	56–223; 427–573
*ND4*	10,131	11,508	1,378	forward	ATG	TAA	partial	30–417
*ND4L*	9,841	10,137	297	forward	ATG	TAA	complete	
*ND3*	9,421	9,768	348	forward	ATC	TAG	partial	50–105
*COX3*	8,569	9,352	784	forward	–	TAA	partial	start–53
*ATP6*	7,890	8,570	681	forward	ATG	–	partial	129–end
*ATP8*	7,729	7,932	204	forward	ATG	TAA	complete	
*COX2*	6,978	7,661	684	forward	ATG	TAA	partial	44–151
*COX1*	5,302	6,840	1,539	forward	ATG	TAA	partial	158–235
*ND2*	3,891	4,925	1,035	forward	ATT	TAA	partial	46–144; 234–312
*ND1*	2,727	3,681	955	forward	GTG	TAG	complete	

**Note:**

Start, the first position along α strand; Stop, the last position along α strand; Length, the size of the sequence; fcd, first codon; scd, stop codon. For partial gene sequences, missing fragments are indicated in the last column.

### Relative synonymous codon usage

**Figure 2:**
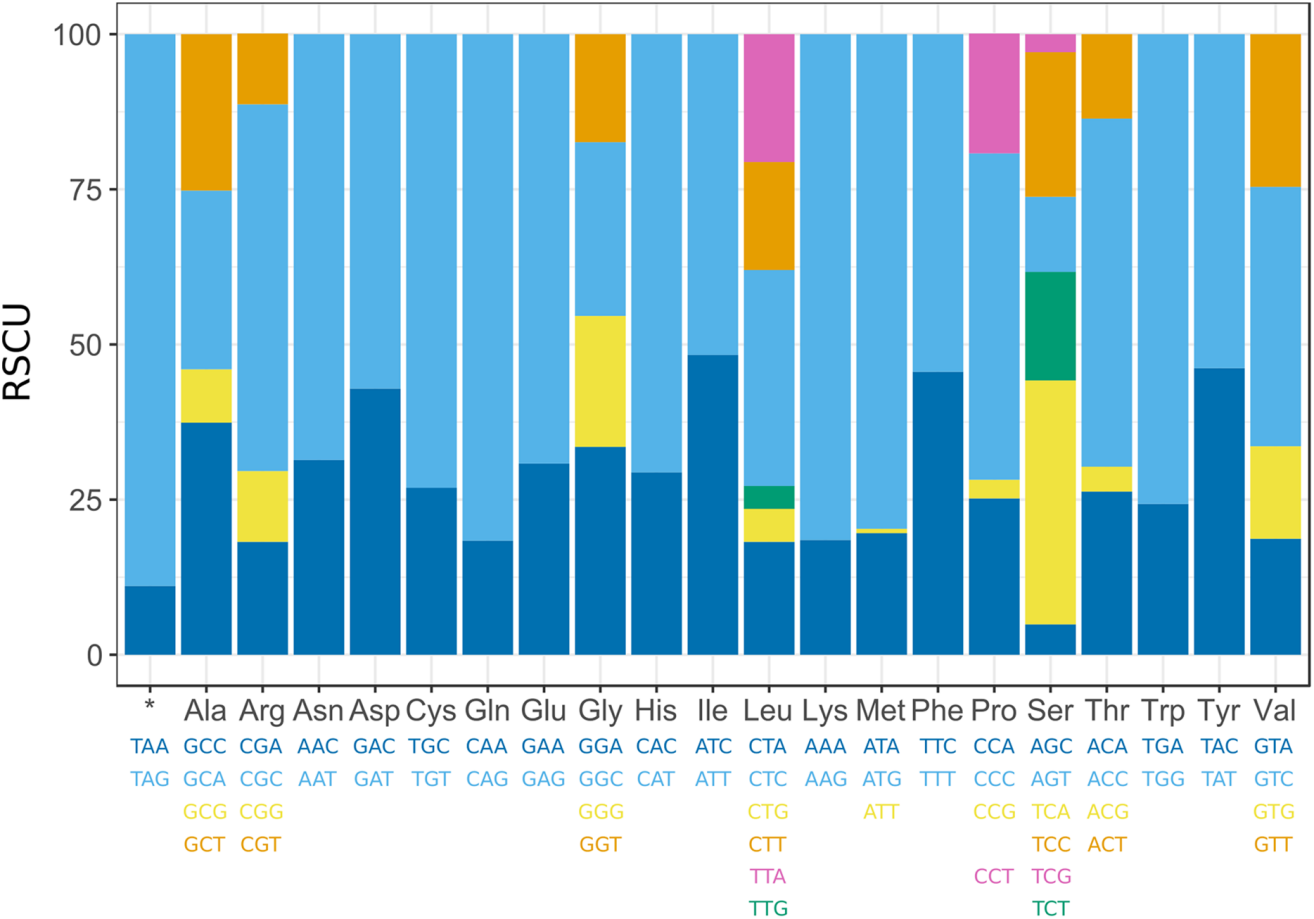
Relative synonymous codon usage (RSCU) in mitochondrial protein-coding genes of *Hyperacrius fertilis*.

The initial codons for 13 PCGs of *H. fertilis* were the canonical putative start codons ATN (ATG for *COX1*, *COX2*, *ATP8*, *ATP6*, *ND4l*, *ND4*, *ND6* and *CYTB*; ATT for *ND2*; ATA for *ND5* and ATC for *ND3*). *ND1* starts with GTG. The typical termination codon (TAA or TAG) occurs in all PCGs. The codon usage pattern of *H. fertilis* mitogenome is shown in Fig. 2. In mtDNA protein-coding genes Arg, Gly, Pro, Thr and Val are varied in codons. Leu and Ser turned out to be the most diverse with the frequency of CTA (34.8%), CTC (18.2%), CTG (5.3%), CTT (17.4%), TTA (20.6%), TTG (3.7%) for Leu and AGC (12.1%), AGT (4.9%), TCA (39.3%), TCC (23.3%), TCG (2.9%) and TCT (17.5%) for Ser, respectively. The substitution frequency in each codon position for *H. fertilis* mitogenome matches with frequency of other analyzed in this study Arvicolinae subfamily mitogenomes (Fig. S3).

### Phylogenetic analyses

Disparity index test calculated using MEGA X revealed significant a variation of patterns of nucleotide substitutions between analyzed sequences when all three codon positions in alignment were considered. Disparity index test yielded in very similar results for two datasets, consisting of alignment with 3rd codon position masked by RY-coding or completely removed (Table S4). Both alignments included variation in patterns of nucleotide substitutions, but the number of disparities was remarkably lower than in a complete dataset. According to IQ-TREE, all sequences in the dataset pass the chi-square nucleotide composition test when only 1st and 2nd codon positions were included in the alignment. In both alignments where all three codon positions were considered and where 3rd position was masked by RY-coding, 7 sequences did not pass the chi-square test, including *H. fertilis* (Table S5).

**Figure 3:**
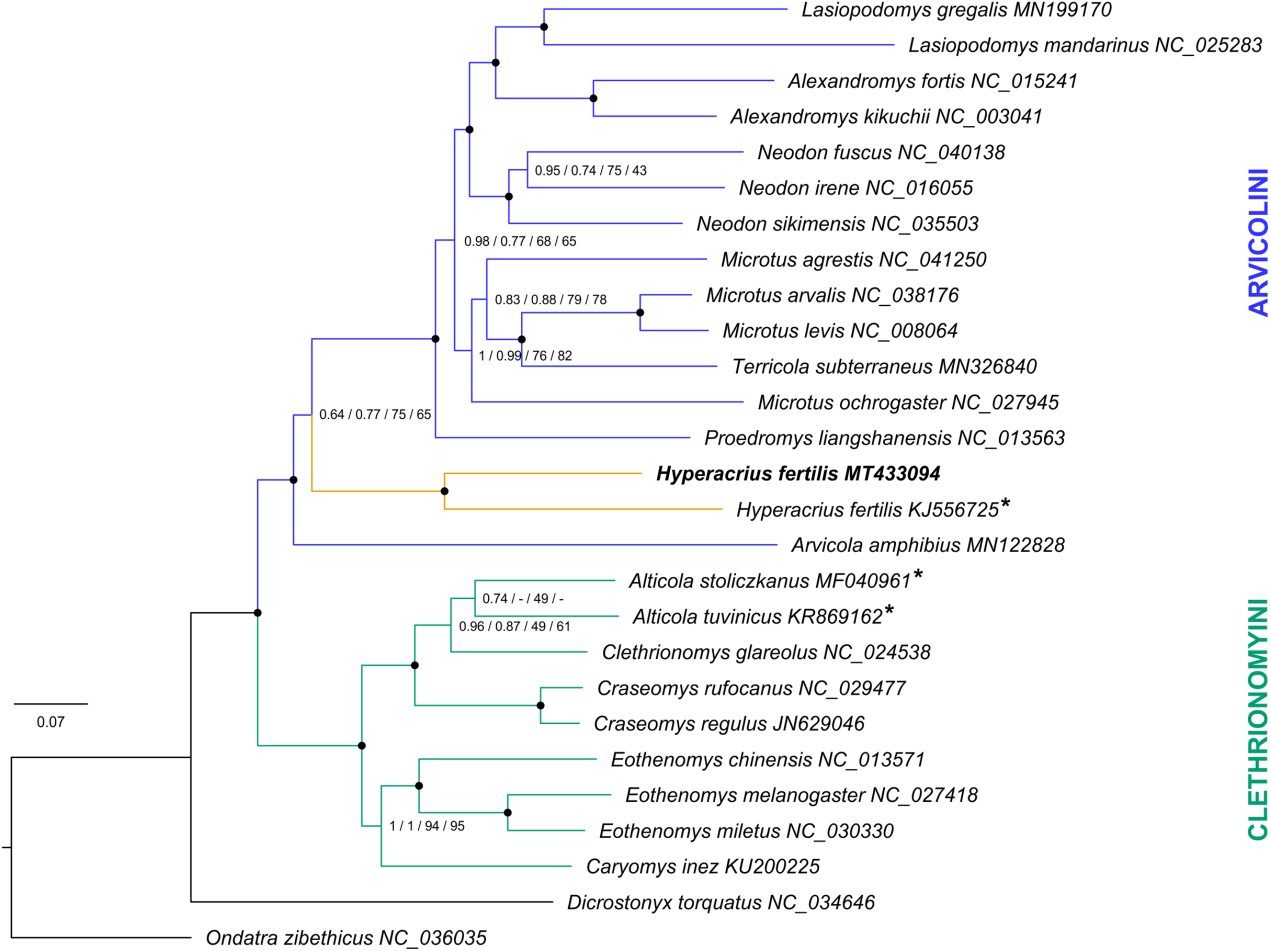
Bayesian and Maximum likelihood phylogenetic reconstruction of tribes Arvicolini and Clethrionomyini using mitogenomes. The trees were inferred from the concatenated dataset of 13 mitochondrial protein-coding genes. The taxa titles include the NCBI accession numbers. For full information on assembled mitochondrial genome sequences used in the study see Table S1. Cytochrome b sequences are marked with asterisk. Node labels display the following supports: BI complete/BI RY-coded 3rd codon position/ML complete/ML RY-coded 3rd codon position. Black circles show nodes with 0.95-1.0 BI and/95-100 ML support.

Phylogenetic relationships based on the nucleotide sequences of the 13 PCGs were obtained with BI and ML analyses (Fig. 3). Both analyses produced trees of identical topology for the complete alignment and the alignment with RY-coded 3rd codon position. The clades respective to major tribes Arvicolini and Clethrionomyini were robustly supported. The newly sequenced mitochondrial genome of *Hyperacrius fertilis* and previously published *CYTB* sequence of *H. fertilis* (GenBank accession KJ556725) formed a significantly supported clade (100% Bayesian probability and 96% ultrafast bootstrap support for complete alignment). Both BI and ML analyses thus showed that neither sequence of mitochondrial genome nor *CYTB* sequence of *H. fertilis* clustered with *CYTB* sequences of *Alticola* (Fig. 3). Phylogenetic reconstructions based on the alignment with removed 3rd codon position showed no statistical support for either Arvicolini and Clethrionomyini clades, or the position of *Hyperacrius fertilis* (Fig. S3). Mitochondrial genome sequence of *H. fertilis* clustered with Arvicolini with no support, the *CYTB* sequence of *H. fertilis* grouped with Clethrionomyini and did not form a statistically supported clade with *Alticola*.

Separate gene trees (Fig. S4) with rare exceptions show polytomy or clusters with low supports due to their low variability, some genes (*COX3*, *ND5*) give a picture similar to that obtained when analyzing complete alignment. The *CYTB* tree constructed from the alignment where the previously published sequence of *H. fertilis* (KJ556725) and other *CYTB* sequences used by Kohli et al. (2014) were added (Fig. S4), demonstrated that Arvicolini does not form monophyletic clade due to the position of *Arvicola* at the base of the tree, *Hyperacrius* forms its own branch in the polytomy.

### Cranial morphology and dentition of the studied *H. fertilis* museum specimen

**Figure 4:**
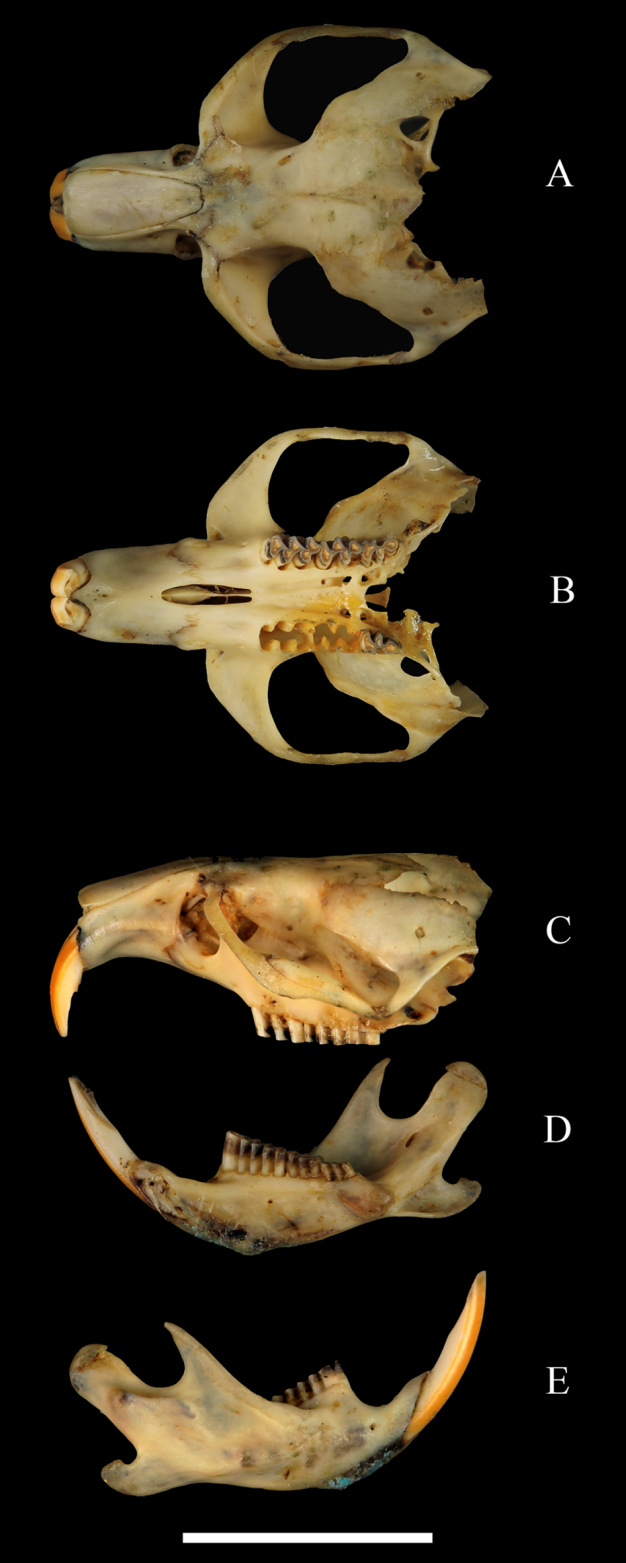
*Hyperacrius fertilis brachelix* ZIN No. 29256, skull and lower jaw. (A) Dorsal view. (B) Bottom view. (C) Lateral view. (D) Right mandible, lingual side. (E) Labial side. Ruler = 1 cm.

**Figure 5:**
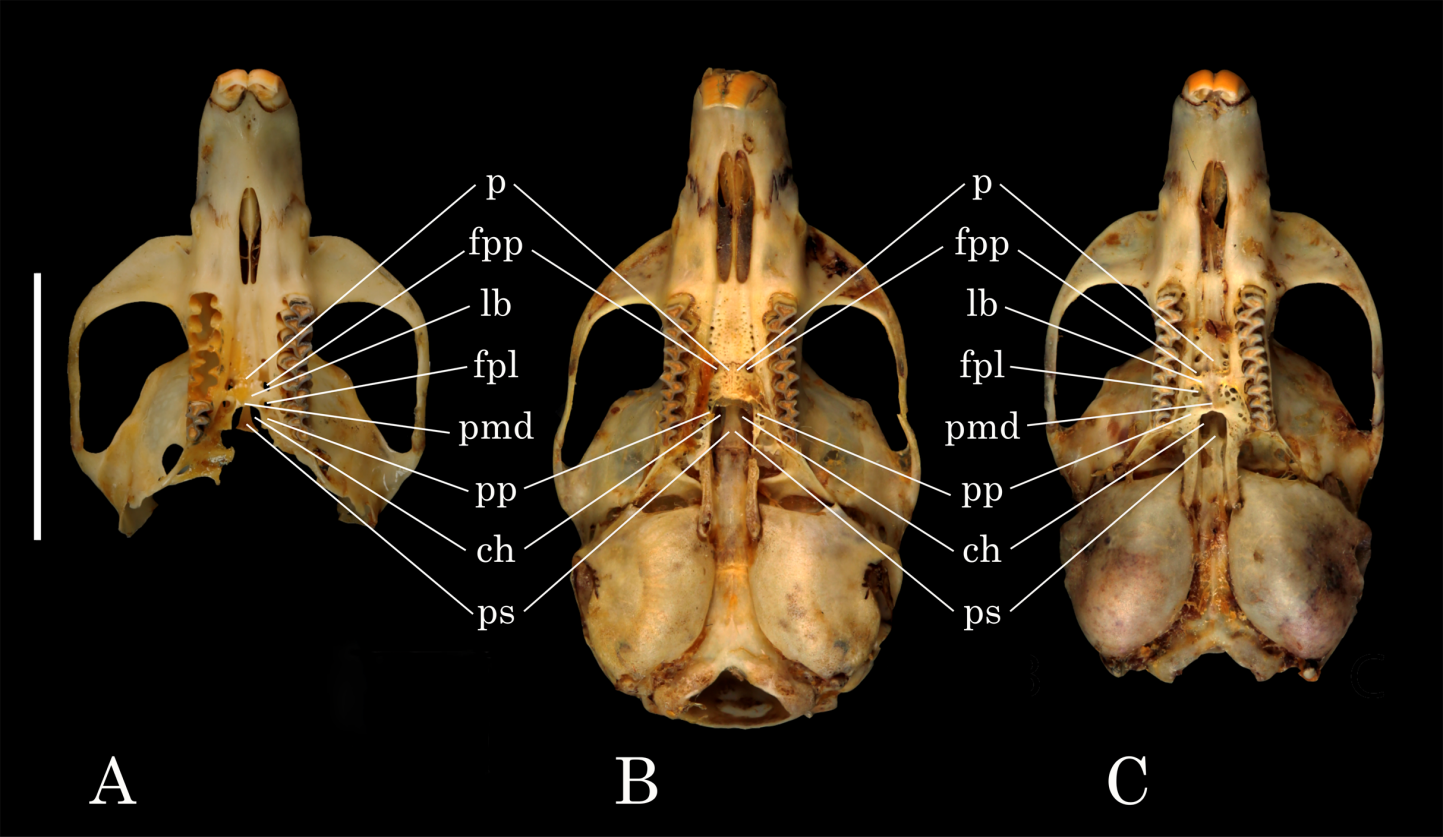
Comparative hard palate morphology in *Hyperacrius*, *Alticola* and *Microtus*. Different types of hard palate: p, palate; fpp, *foramen palatinum posterior*; lb, lateral bridge; fpl, *fossa palatina lateralis* (posterolateral palatal pits); pmd, *protuberantia marginalis descendens* (medial sloping protrusion); pp, *processus pyramidalis*; ch, choanae (*fossa mesopterygoidea*); ps, *praesphenoideum*: (A) *Hyperacrius fertilis brachelix* ZIN No. 29256—“microtini-type” palate; (B) *Alticola argentatus* ZIN No. 74245—“clethrionomyini-type” palate; (C) *Microtus socialis* No 77664—“microtini-type”. Ruler = 1 cm.

The skull dimensions of the studied specimen were the following: diastema length—7.7 mm; zygomatic breadth—13.9 mm; intraorbital breadth—4.0 mm; length of incisive foramina—3.0 mm; length of nasals—6.3 mm; length of the lower jaw—13.8 mm; length of upper molar row—5.6 mm; length of lower molar row—5.6 mm; length of upper molar M3—1.10 mm; length of lower molar m1—2.75 mm. Compared to the published measurements of other representatives of *Hyperacrius* (Phillips, 1969), this specimen can be considered as a subadult specimen. The ridges of the skull (Fig. 4) are poorly pronounced. Interorbital ridge (*crista frontalis*) is only slightly outlined. Postorbital process (*tuber postorbitalis*) is small. The examined skull was highly damaged, yet the presphenoid in the form of a narrow plate ending at the level of the posterior lobe of the upper molar M3, was clearly notable (Fig. 5A). The skull of *Hyperacrius* that was examined had clearly a “microtini” type of hard palate structure with a short and wide median sloping protrusion, and the lateral bridge is well-expressed.

**Figure 6:**
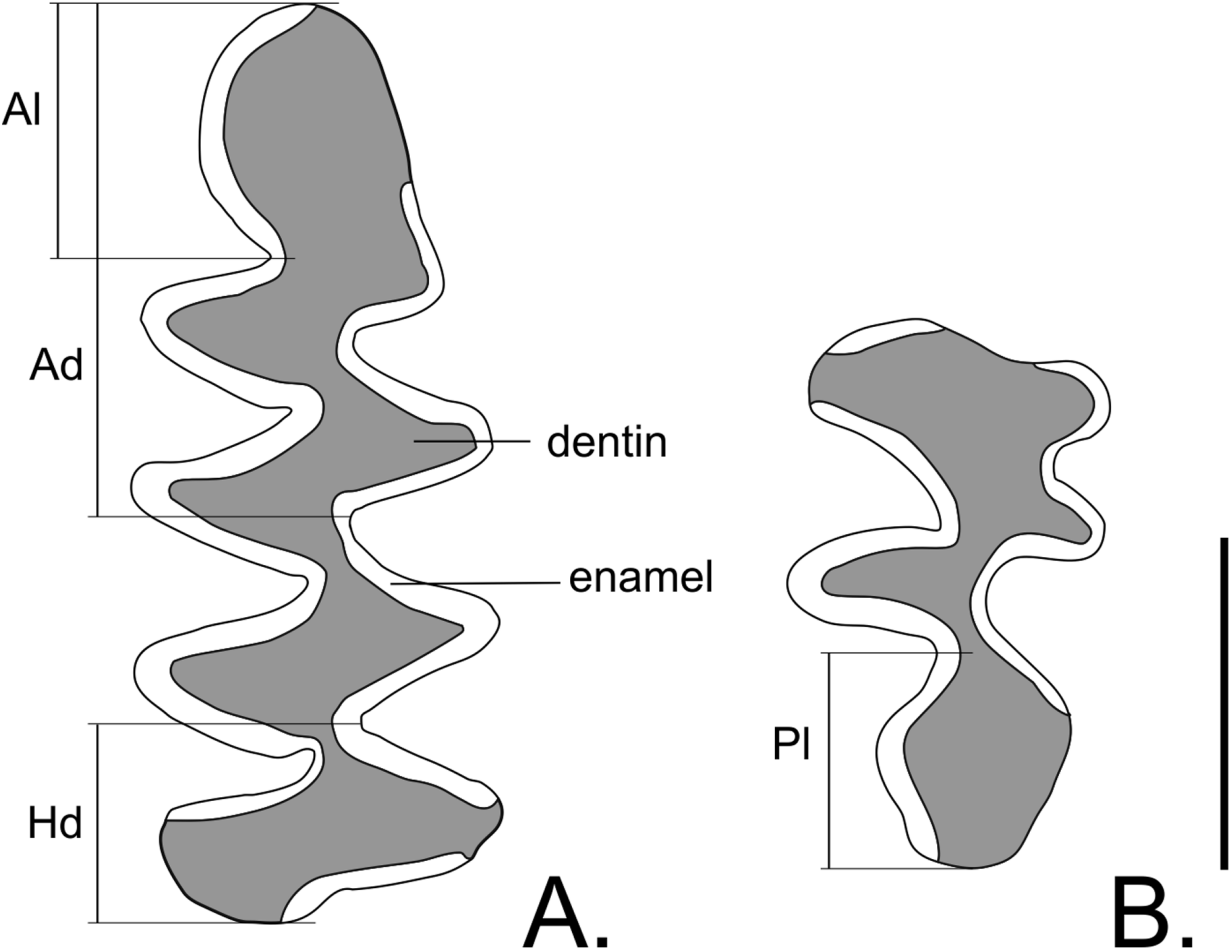
Molars of *Hyperacrius fertilis brachelix* ZIN No. 29256. (A) Molar m1. (B) Molar M3. Ad, antericonid; Al, anterior lobe; Hd, hypoconid; Pl, posterior lobe. Ruler = 1 mm.

Despite the dentition of *Hyperacrius* was thoroughly described by Hinton (1926), taking in account the importance of these characters for systematics, we provide here the short description of molars with the special attention to the structure of first lower molar m1 and third upper molar M3. Molars in *H. fertilis* are rootless with slightly pronounced positive differentiation, that is, enamel is thicker on the anterior edge of the triangles than on the posterior edges (see Martin (1987) for the description). All three molars within the lower jaw have the opposing arrangement of salient angles, and their length at the inner and outer molar sides is equal. Lingual side of m1 has four re-entrant angles and the labial side has three (Fig. 6). The upper molars have an alternating arrangement of salient angles. The upper third molar (M3) has two re-entrant angles from both the lingual and labial sides (Fig. 6). A similar pattern of M3 is typical also for *Alticola*.

## Discussion

### General features of the Hyperacrius mitochondrial genome

Our data show that the mitochondrial genome of *H. fertilis* is characterized by the typical order and direction of protein-coding genes, ribosomal RNA genes, tRNA genes and the putative control region, consistent with other vole and rodent taxa (Reyes et al., 2000; Horn et al., 2011; Bendová et al., 2016; Cao et al., 2016; Folkertsma et al., 2018, Bondareva & Abramson, 2019). In our assembly of *H. fertilis* eight PCGs were incomplete (*ND2-5, COX1-3, ATP6*), which is expected as the consequence of challenges of DNA extraction and sequencing from old dry museum specimens (e.g., Roca et al., 2004; Sato et al., 2016 and references therein). We confirm the authenticity of the studied specimen by analysis of historic DNA damage, comparison of topologies of trees reconstructed for each PCG and calculating of substitution frequency in each codon position.

### Review of *H. fertilis* distinctive morphological features

The taxonomic and phylogenetic position of the genus *Hyperacrius* has always been remaining obscure due to the unique combination of primitive and advanced morphological features that distinguish *Hyperacrius* from both Clethrionomyini and Arvicolini. At the same time, this unusual combination of primitive and advanced characters was the strongest argument for substantiating the separate taxonomic status of the subgenus (Miller, 1896) and genus rank (Hinton, 1926; Gromov & Polyakov, 1977 and others).

The structure of the palate is of special diagnostic importance within the subfamily Arvicolinae (Ognev, 1948; Gromov & Polyakov, 1977; Pozdnyakov, 2008). Three main types of hard palate structure have been distinguished: (1) the palate typical for *Clethrionomys-Alticola* (“clethrionomyini type”), (2) for *Microtus-Arvicola* (“microtini”) and (3) type typical only for *Dinaromys* Kretzoi, 1955 (Zazhigin, 1980). The first (“clethrionomyini type”) type palate (Fig. 5B) is characterized by the absence of a medial sloping protrusion (*protuberantia marginalis descendens*); as a result, the palate abruptly terminates near the choanae (*fossa mesopterygoidea*) and overhangs the posterolateral palatal pits (*fossa palatina lateralis*).

The *Microtus-Arvicola* (“microtini” type) differs by the pronounced medial sloping protrusion, and the horizontal edge of the palate smoothly connects with the inner edges of interior pyramidal processes, *foramen palatinum posterior* and *fossa palatina lateralis* are well-pronounced and separated by a bony lateral bridge (Fig. 5C).

The major revision of Arvicolinae by Hinton (1926), presents the morphological description of *Hyperacrius* with both species—*H. wynnei* and *H. fertilis* analyzed. The description shows that *H. wynnei* distinctly has the hard palate of “clethrinomyini” type. The Fig. 93 (p.330) in Hinton (1926) presenting the skull *Hyperacrius fertilis brachelix*, demonstrates that the hard palate in this species smoothly goes over till *processus pyramidalis* and *fossa palatina lateralis*, whereas the Fig. 94 (p.331) shows that in *H. wynnei* the posterior edge of the palate sharply ends up at choanae (Hinton, 1926). As a result, Hinton’s description shows that both types of hard palate structure can be observed among species of genus *Hyperacrius*. Such polymorphic state character is typical for the representatives of the extinct genus *Mimomys* Forsyth Major, 1902 *sensu lato* (Zazhigin, 1980).

While the analyzed skull of *H. fertilis* was incomplete (Figs. 4 and 5), we can clearly confirm that the hard palate (Fig. 5A) was found to be more of “microtini” type or “palate normal” according to Miller (1896).

Most morphological features characteristic for the genus *Hyperacrius* and the studied specimen of *H. fertilis brachelix* distinguish them from both tribes Cletherionomyini and Arvicolini and present a unique combination of primitive and advanced character states. Among the primitive features is the simple structure of hard palate in *H. wynnei*: median sloping protrusion is absent, that defines the type “clethriomyini”, whereas in *H. fertilis* the palate structure is more advanced: median sloping protrusion, lateral bridge and posterolateral palatal pits are present, that defines the “microtini” type. Both species alongside with most recent Arvicolini have hypsodont rootless teeth (advanced feature) however, molars have a number of very primitive features, that are: cement in the re-entrant angles is absent, salient angles at the lower molars arranged in opposing rather than altering way, m1 has only four salient angles on both lingual and labial sides; M3 has only two salient angles at lingual and labial sides (similar to one in *Alticola*); In *H. fertilis*, enamel thickness is almost equal on anterior and posterior edges of salient angles, most part of re-entrant angles is formed by radial enamel layer and only at the top of the salient angles radial layer is supplemented by lamellar one. The lamellar layer is slight and tangential enamel layer is absent (Koenigswald, 1980). The study of enamel ultrastructure variability in Arvicolinae (Koenigswald, 1980) showed dramatic differences between genera *Hyperacrius* and *Alticola*. Nevertheless, according to the conventional views, *Hyperacrius* is placed closer to *Alticola* (Gromov & Polyakov, 1977; Corbet, 1978; Corbet & Hill, 1992; Carleton & Musser, 2005) and within the tribe Clethrionomyini.

### Phylogenetic position and evolutionary history of Hyperacrius

The phylogenetic analysis based on mitogenomic sequences plausibly shows that *Hyperacrius* represent one of the earliest lineages within the tribe Arvicolini, sister to the main group of genera within the tribe (Fig. 3). This result was supported by BI and ML reconstructions based on the complete alignment of 13 mitochondrial protein-coding genes, as well as on the alignment where third codon position was masked by RY-coding for purines and pyrimidines respectively. The approach of excluding third codon position or mask it with RY-coding is often considered in phylogenetic reconstructions in order to control for potential biasing effects of base compositional heterogeneity and saturation in 3rd position transitions (Chang & Campbell, 2000; Fenn et al., 2008; Breinholt & Kawahara, 2013; Chen et al., 2018; Wang et al., 2019). We thus also performed BI and ML analysis from the alignment where the third codon position was removed (Fig. S3). This analysis provided no support for the position of *Hyperacrius* and also did not provide support for major clades representing tribes Arvicolini and Clethrionomyini, and thus the approach of removing third codon position seems to be irrelevant in studies of mitogenomes of Arvicolinae.

The divergence between the *Hyperacrius* and the main stem of Arvicolini occurs right after the deviation of *Arvicola amphibius* and thus, *Hyperacrius* represents an evolutionary lineage considering to be independent of tribe “Microtini”. This result of phylogenetic analysis thus clearly indicates that morphological features similar in *Hyperacrius* and *Alticola* and other genera of Clethrionomyini are convergent. Thus, *Hyperacrius* and *Alticola*, in contrast to the conventional views (Hinton, 1926; Corbet, 1978; Corbet & Hill, 1992), have no recent common ancestors. The first molecular study of *Hyperacrius* based on sequencing of cytochrome *b* gene fragment (Kohli et al., 2014) also resulted in the assumption that *H. fertilis* presumably is not a member of Clethrionomyini clade and its phylogenetic and systematic position should be comprehensively reviewed. A phylogenetic analysis of morphological features across Arvicolinae also found *Hyperacrius* to be closer to subterranean arvicolids, such as *Prometheomys* Satunin, 1901 and *Ellobius* Fischer, 1814 (Robovský, Řičánková & Zrzavý, 2008), rather than to *Alticola* (Phillips, 1969). Since numerous cases of morphological parallelism in skull and dentition structures within the subfamily Arvicolinae are known (Shevyreva, 1976; Chaline et al., 1999), the morphological similarity between *Alticola* and *Hyperacrius*, both inhabiting highland habitats, most likely convergent.

Our phylogenetic reconstruction based on mitochondrial genomes shows that *Hyperacrius* represents one of the basal branches of the tribe Arvicolini yet no direct ancestors of this genus are known. It is generally accepted that the direct ancestors of recent forms of Arvicolini were extinct voles grouped into genus *Mimomys*. This group is characterized by rooted, cementless molars with the enamel thicker on the posterior edge of the triangles than on the anterior one (negative differentiation, Martin, 1987) and the more complex enamel ultrastructure with three distinct layers. Teeth of *Mimomys* are with alternating triangles both at the upper and lower molars (Gromov & Polyakov, 1977; Chaline & Graf, 1988; Topachevsky & Nesin, 1989; Agadzhanian, 2009; Fejfar et al., 2011 and references therein). Further evolutionary transformations of Arvicoline dentition during the Pliocene-Pleistocene are traced in the paleontological record and are related to the so-called “allophaiomys” stage, featured by hypsodonty, loss of molar roots, and an contrasting pattern of distribution of enamel thickness and to a variable extent of enamel differentiation. Some recent representatives of the tribe Arvicolini retained the “allophaiomys” type of molar structure (Martin & Tesakov, 1998; Golenishchev & Malikov, 2006). This process of dentition transformation from the so-called “mimomys” stage through “allophaiomys” to “microtus” took place independently in various lineages of the Arvicolini tribe. Among all known forms of *Mimomys sensu lato*, there are none with such primitive dentition characters as in *Hyperacrius* (Gromov & Polyakov, 1977; Zheng & Li, 1986; Chaline et al., 1999; Rabeder, 1981; Topachevsky & Nesin, 1989; Agadzhanian, 2009; Fejfar et al., 2011). Most likely, the origin and evolution of genus *Hyperacrius* was closely associated with the early transition to mostly subterranean life and related to this secondary simplification of the molar pattern, analogous to those observed in the evolution of the highly specialized subterranean genus *Ellobius* (Abramson et al., 2009).

### Taxonomic implications

The results of phylogenetic analysis based on the concatenated sequences of 13 mitochondrial PCGs altered the conventional view on the phylogenetic relatedness of *Hyperacrius* to *Alticola* and prompted the revision of morphological characters underlying the former assumption. Both molecular and morphological data demonstrate that the genus *Hyperacrius* should not be considered within the tribe Clethrionomyini. Mitochondrial genome sequencing shows that *Hyperacrius* can plausibly be regarded as belonging to the tribe Arvicolini, yet more studies involving nuclear genes are required to test this hypothesis.

## Conclusions

Our study shows that the century-old dry specimens could be remarkably helpful for the phylogenetic reconstructions. Using shotgun sequencing, we obtained the mitogenome of Subalpine Kashmir vole *Hyperacrius fertilis*, containing nearly all mitochondrial protein-coding genes sequences. Phylogenetic analyses of the obtained sequences in combination with mitogenomic data available for species of the tribes Arvicolini and Clethrionomyini and examination of key distinctive morphological features assume a plausible phylogenetic position of *H. fertilis* within the tribe of Arvicolini, supposedly representing one of the earliest diverging lineages. Our results substantiate the necessity for reconsidering the conventional classification as those presented in the recent reference book on mammals (Carleton & Musser, 2005). Nevertheless, further research, including either transcriptome or complete genome sequencing, preferably implemented for newly collected specimens, would be crucial for more accurate estimates of the evolutionary history of this enigmatic species from Kashmir. It is also worth mentioning that further molecular studies on *H. wynnei* are required for the accurate revision of the taxonomic position of the genus *Hyperacrius*. Since the two species of the genus demonstrate remarkable morphological differences, genetic differentiation among the species of the genus could be also pronounced. Considering that the *H. fertilis* is a type species within the genus *Hyperacrius*, the current level of knowledge is sufficient for altering the conventional view on its phylogenetic affinities and corresponding on taxonomic position within the Arvicolinae subfamily.

## Supplemental Information

10.7717/peerj.10364/supp-1 Supplemental Information 1 Nucleotide misincorporations at 5’-termini (A) and 3’-termini (B) of the *Hyperacrius fertilis* calculated using mapDamage. All possible misincorporations are plotted in gray, except for guanine to adenine (G>A, blue lines) and cytosine to thymine (C>T, red lines).

10.7717/peerj.10364/supp-2 Supplemental Information 2 Mapping of raw Illumina reads of *Hyperacrius fertilis* against the reference mitochondrial genome of *Clethrionomys glareolus* using Geneious Prime.

10.7717/peerj.10364/supp-3 Supplemental Information 3 Phylogenetic reconstruction of tribes Arvicolini and Clethrionomyini using alignment with excluded third codon position. The trees were inferred from the concatenated dataset of 13 mitochondrial protein-coding genes. Node labels display BI/ML support; black circles show nodes with 0.95-1.0/95-100 support. Cytochrome *b* sequences are marked with asterisk.

10.7717/peerj.10364/supp-4 Supplemental Information 4 Bayesian phylogenetic reconstruction of tribes Arvicolini and Clethrionomyini inferred separately from alignments of 13 mitochondrial protein-coding genes. Bayesian trees inferred from separate PCGs partitioned by codon position. Node labels display BI posterior probabilities (PP).

10.7717/peerj.10364/supp-5 Supplemental Information 5 Information regarding the taxa included in this study.

10.7717/peerj.10364/supp-6 Supplemental Information 6 Optimal partitioning scheme of the concatenated alignment of mitochondrial protein-coding genes obtained using PartitionFinder for the subsequent Bayesian and Maximum Likelihood phylogenetic analyses.

10.7717/peerj.10364/supp-7 Supplemental Information 7 *Hyperacrius fertilis* mitochondrial genome annotation obtained using the MITOS web server and verified using Geneious Prime.

10.7717/peerj.10364/supp-8 Supplemental Information 8 Test of the homogeneity of substitution patterns between sequences (see 2nd and 3rd sheet for other dataset results).

10.7717/peerj.10364/supp-9 Supplemental Information 9 IQTREE chi-square nucleotide composition tests.

10.7717/peerj.10364/supp-10 Supplemental Information 10 Mitogenome sequence of Hyperacrius fertilus available at GenBank.

## Additional Information and Declarations

### DNA Deposition

The following information was supplied regarding the deposition of DNA sequences: The newly determined mitogenome sequence of *Hyperacrius fertilus* is available in GenBank: No. MT433094.

### Data Availability

The following information was supplied regarding data availability: A dataset containing the concatenated alignment of 13 protein-coding genes is available at GitHub: https://github.com/ZaTaxon/Hyperacrius.

## References

[ref-1] Abramson NI, Lebedev VS, Tesakov AS, Bannikova AA (2009). Supraspecies relationships in the subfamily Arvicolinae (Rodentia, Cricetidae): an unexpected result of nuclear gene analysis. Molecular Biology.

[ref-2] Agadzhanian AK (2009). Small mammals of Pliocene-Pleistocene of the Russian plain.

[ref-3] Agrawal VC (2000). Taxonomic studies on Indian muridae and hystricidae (Mammalia, Rodentia). Records of the Zoological Survey of India/Occasional Paper.

[ref-4] Andrews S (2010). FastQC: a quality control tool for high throughput sequence data. http://www.bioinformatics.babraham.ac.uk/projects/fastqc.

[ref-5] Bankevich A, Nurk S, Antipov D, Gurevich AA, Dvorkin M, Kulikov AS, Lesin VM, Nikolenko SI, Pham S, Prjibelski AD, Pyshkin AV, Sirotkin AV, Vyahhi N, Tesler G, Alekseyev MA, Pevzner PA (2012). SPAdes: a new genome assembly algorithm and its applications to single-cell sequencing. Journal of Computational Biology.

[ref-6] Bendová K, Marková S, Searle JB, Kotlík P (2016). The complete mitochondrial genome of the bank vole Clethrionomys glareolus (Rodentia: Arvicolinae). Mitochondrial DNA Part A.

[ref-7] Bernt M, Donath A, Jühling F, Externbrink F, Florentz C, Fritzsch G, Pütz J, Middendorf M, Stadler PF (2013). MITOS: improved de novo metazoan mitochondrial genome annotation. Molecular Phylogenetics and Evolution.

[ref-8] Bondareva OV, Abramson NI (2019). The complete mitochondrial genome of the common pine vole Terricola subterraneus (Arvicolinae, Rodentia). Mitochondrial DNA Part B.

[ref-9] Breinholt JW, Kawahara AY (2013). Phylotranscriptomics: saturated third codon positions radically influence the estimation of trees based on next-gen data. Genome Biology and Evolution.

[ref-10] Cao W, Xia Y, Dang X, Xu Q (2016). The first complete mitochondrial genome of the Microtus ochrogaster. Mitochondrial DNA Part A.

[ref-11] Carleton MD, Musser GG, Wilson DE, Reeder DM (2005). Order rodentia, mammal species of the world: a taxonomic and geographic reference.

[ref-12] Chaline J, Brunet-Lecomte P, Montuire S, Viriot L, Courant F (1999). Anatomy of the arvicoline radiation (Rodentia): palaeogeographical, palaeoecological history and evolutionary data. Annales Zoologici Fennici.

[ref-13] Chaline J, Graf J-D (1988). Phylogeny of the Arvicolidae (Rodentia): biochemical and paleontological evidence. Journal of Mammalogy.

[ref-14] Chang BS, Campbell DL (2000). Bias in phylogenetic reconstruction of vertebrate rhodopsin sequences. Molecular Biology and Evolution.

[ref-15] Chen ZT, Zhao MY, Xu C, Du YZ (2018). Molecular phylogeny of Systellognatha (Plecoptera: Arctoperlaria) inferred from mitochondrial genome sequences. International Journal of Biological Macromolecules.

[ref-16] Cock PJ, Antao T, Chang JT, Chapman BA, Cox CJ, Dalke A, Friedberg I, Hamelryck T, Kauff F, Wilczynski B, De Hoon MJ (2009). Biopython: freely available Python tools for computational molecular biology and bioinformatics. Bioinformatics.

[ref-17] Corbet GB (1978). The mammals of the Palaearctic region: a taxonomic review.

[ref-18] Corbet GB, Hill JE (1992). The mammals of the Indomalayan region: a systematic review.

[ref-19] Darling AC, Mau B, Blattner FR, Perna NT (2004). Mauve: multiple alignment of conserved genomic sequence with rearrangements. Genome Research.

[ref-20] Fejfar O, Heinrich W-D, Kordos L, Maul LC (2011). Microtoid cricetids and the early history of arvicolids (Mammalia, Rodentia). Palaeontologia Electronica.

[ref-21] Fenn JD, Song H, Cameron SL, Whiting MF (2008). A preliminary mitochondrial genome phylogeny of Orthoptera (Insecta) and approaches to maximizing phylogenetic signal found within mitochondrial genome data. Molecular Phylogenetics and Evolution.

[ref-22] Folkertsma R, Westbury MV, Eccard JA, Hofreiter M (2018). The complete mitochondrial genome of the common vole, Microtus arvalis (Rodentia: Arvicolinae). Mitochondrial DNA Part B.

[ref-23] Golenishchev FN, Malikov VG (2006). The developmental conduit of the tribe Microtini (Rodentia, Arvicolinae): systematic and evolutionary aspects. Russian Journal of Theriology.

[ref-24] Gromov IM, Polyakov IYa (1977). Fauna SSSR: Mammals. Voles (Microtinae).

[ref-25] Hinton MAC (1926). Monograph of the voles and lemmings (Microtinae) living and extinct.

[ref-26] Hoang DT, Chernomor O, Von Haeseler A, Minh BQ, Vinh LS (2018). UFBoot2: improving the ultrafast bootstrap approximation. Molecular Biology and Evolution.

[ref-27] Hooper ET, Hart BS (1962). A synopsis of recent North American microtine rodents.

[ref-28] Horn S, Durka W, Wolf R, Ermala A, Stubbe A, Stubbe M, Hofreiter M (2011). Mitochondrial genomes reveal slow rates of molecular evolution and the timing of speciation in beavers (Castor), one of the largest rodent species. PLOS ONE.

[ref-29] Jónsson H, Ginolhac A, Schubert M, Johnson PL, Orlando L (2013). mapDamage2.0: fast approximate Bayesian estimates of ancient DNA damage parameters. Bioinformatics.

[ref-30] Katoh K, Misawa K, Kuma K, Miyata T (2002). MAFFT: a novel method for rapid multiple sequence alignment based on fast Fourier transform. Nucleic Acids Research.

[ref-31] Koenigswald WV (1980). Schmelzstrukture und Morphologie in den Molaren der Arvicolidae (Rodentia). Abhandlungen der Senckenbergischen Naturforschenden Gesellschaft.

[ref-32] Köchl S, Niederstätter H, Parson W (2005). DNA extraction and quantitation of forensic samples using the phenol-chloroform method and real-time PCR. Methods in Molecular Biology.

[ref-33] Kohli BA, Speer KA, Kilpatrick CW, Batsaikhan N, Damdinbaza D, Cook JA (2014). Multilocus systematics and non-punctuated evolution of Holarctic Myodini (Rodentia: Arvicolinae). Molecular Phylogenetics and Evolution.

[ref-34] Krystufek B, Nesakov AS, Lebedev VS, Bannikova AA, Abramson NI, Shenbrot G (2020). Back to the future: the proper name for red-backed voles is Clethrionomys Tilesius and not Myodes Pallas. Mammalia.

[ref-35] Kumar S, Gadagkar SR (2001). Disparity index: a simple statistic to measure and test the homogeneity of substitution patterns between molecular sequences. Genetics.

[ref-36] Kumar S, Stecher G, Li M, Knyaz C, Tamura K (2018). MEGA X: molecular evolutionary genetics analysis across computing platforms. Molecular Biology and Evolution.

[ref-37] Lanfear R, Frandsen PB, Wright AM, Senfeld T, Calcott B (2017). PartitionFinder 2: new methods for selecting partitioned models of evolution for molecular and morphological phylogenetic analyses. Molecular Biology and Evolution.

[ref-38] Martin RA (1987). Notes on the classification and evolution of some North American fossil *Microtus*. Journal of Vertebrate Paleontology.

[ref-39] Martin RA, Tesakov A (1998). Introductory remarks: does Allophaiomys exist?. Early Evolution of Microtus: Paludicola.

[ref-40] Miller GS (1896). Genera and subgenera of voles and lemmings. North American Fauna.

[ref-41] Molto JE, Loreille O, Mallott EK, Malhi RS, Fast S, Daniels-Higginbotham J, Marshall C, Parr R (2017). Complete mitochondrial genome sequencing of a burial from a Romano–Christian cemetery in the Dakhleh Oasis, Egypt: preliminary indications. Genes.

[ref-42] Ognev SI (1948). Wild animals of the USSR and adjoining countries.

[ref-43] Pardiñas UFJ, Myers P, León-Paniagua L, Ordóñez Garza N, Cook JA, Kryštufek B, Haslauer R, Bradley RD, Shenbrot GI, Patton JL, Wilson DE, Lacher TEJ, Mittermeier RA (2017). Family Cricetidae (true hamsters, voles, lemmings and new world rats and mice). Handbook of the Mammals of the World. Rodents II.

[ref-44] Phillips CJ (1969). Review of central Asian voles of the genus Hyperacrius, with comments on zoogeography, ecology, and ectoparasites. Journal of Mammalogy.

[ref-45] Pozdnyakov AA (2008). The bony palate morphology in Arvicolinae (Rodentia: Cricetidae), with comments on taxonomy and nomenclature. Sbornik trudov Zoologicheskogo muzeya MGU.

[ref-46] Rabeder G (1981). Die Arvicoliden (Rodentia, Mammalia) aus dem Pliozän und dem älteren Pleistozän von Niederösterreich.

[ref-47] Rambaut A, Drummond AJ, Xie D, Baele G, Suchard MA (2018). Posterior summarization in Bayesian phylogenetics using Tracer 1.7. Systematic Biology.

[ref-48] Reyes A, Gissi C, Pesole G, Catzeflis FM, Saccone C (2000). Where do rodents fit? Evidence from the complete mitochondrial genome of Sciurus vulgaris. Molecular Biology and Evolution.

[ref-49] Robovský J, Řičánková V, Zrzavý J (2008). Phylogeny of Arvicolinae (Mammalia, Cricetidae): utility of morphological and molecular data sets in a recently radiating clade. Zoologica Scripta.

[ref-50] Roca AL, Bar-Gal GK, Eizirik E, Helgen KM, Maria R, Springer MS, O’Brien SJ, Murphy WJ (2004). Mesozoic origin for west Indian insectivores. Nature.

[ref-51] Ronquist F, Teslenko M, Van der Mark P, Ayres DL, Darling A, Höhna S, Larget B, Liu L, Suchard MA, Huelsenbeck JP (2012). MrBayes 3.2: efficient Bayesian phylogenetic inference and model choice across a large model space. Systematic Biology.

[ref-52] Sato JJ, Ohdachi SD, Echenique-Diaz LM, Borroto-Páez R, Begué-Quiala G, Delgado-Labañino JL, Gámez-Díez J, Alvarez-Lemus J, Nguyen ST, Yamaguchi N, Kita M (2016). Molecular phylogenetic analysis of nuclear genes suggests a Cenozoic over-water dispersal origin for the Cuban solenodon. Scientific Reports.

[ref-53] Shevyreva NS (1976). Paleogene rodents of Asia.

[ref-55] Tesakov AS, Lebedev VS, Bannikova AA, Abramson NI (2010). Clethrionomys Tilesius, 1850 is the valid generic name for red-backed voles and Myodes Pallas, 1811 is a junior synonym of Lemmus Link, 1795. Russian Journal Theriology.

[ref-56] Topachevsky VA, Nesin VA (1989). Rodents of the Moldavian and Khaprovian faunistic complexes of Kotlovina section.

[ref-60] Trifinopoulos J, Nguyen LT, von Haeseler A, Minh BQ (2016). W-IQ-TREE: a fast online phylogenetic tool for maximum likelihood analysis. Nucleic Acids Research.

[ref-57] Wang Y, Cao JJ, Li N, Ma GY, Li WH (2019). The first mitochondrial genome from Scopuridae (Insecta: Plecoptera) reveals structural features and phylogenetic implications. International Journal of Biological Macromolecules.

[ref-58] Zazhigin VS (1980). Late Pliocene and Anthropogene rodents of the South of Western Siberia.

[ref-59] Zheng SH, Li CK (1986). A review of Chinese *Mimomys* (Arvicolidae, Rodentia). Vertebrata Palasiatica.

