# Supplementary material for: Phylogenetic relationships and taxonomic position of genus Hyperacrius (Rodentia: Arvicolinae) from Kashmir based on evidences from analysis of mitochondrial genome and study of skull morphology
Source: PeerJ. 2020 Nov 18;8:e10364. doi: 10.7717/peerj.10364 (PMC7680025; doi:10.7717/peerj.10364)

Figure S1. Nucleotide misincorporations at 5'-termini (A) and 3'-termini (B) of the *Hyperacrius fertilis* calculated using mapDamage.

All possible misincorporations are plotted in gray, except for guanine to adenine (G>A, blue lines) and cytosine to thymine (C>T, red lines).

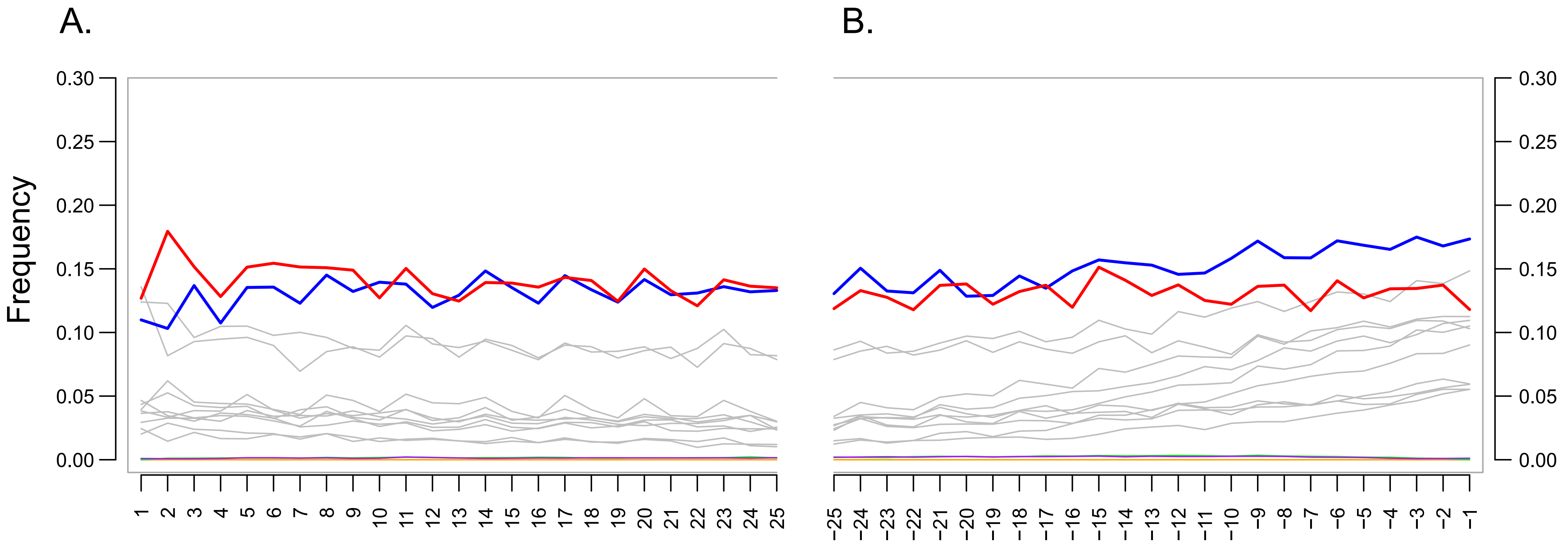

Supplement: Supplemental Information 1 — All possible misincorporations are plotted in gray, except for guanine to adenine (G>A, blue lines) and cytosine to thymine (C>T, red lines). [file peerj-08-10364-s001.pdf]
