# Supplementary material for: Phylogenetic relationships and taxonomic position of genus Hyperacrius (Rodentia: Arvicolinae) from Kashmir based on evidences from analysis of mitochondrial genome and study of skull morphology
Source: PeerJ. 2020 Nov 18;8:e10364. doi: 10.7717/peerj.10364 (PMC7680025; doi:10.7717/peerj.10364)

Figure S2. Mapping of raw Illumina reads of *Hyperacrius fertilis* against the reference mitochondrial genome of *Clethrionomys glareolus* using Geneious Prime.

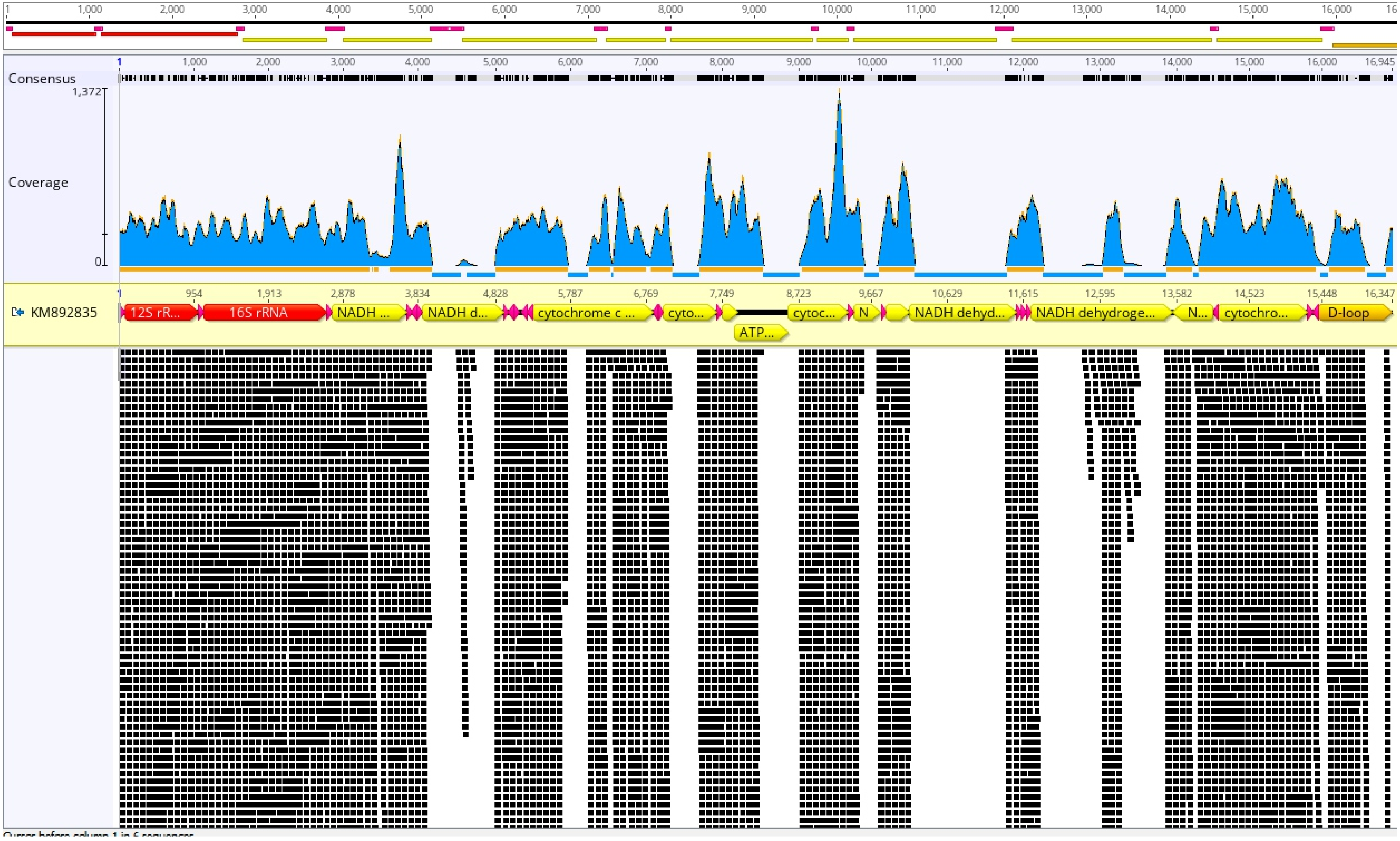

Supplement: Supplemental Information 2 [file peerj-08-10364-s002.pdf]
