# Supplementary material for: Phylogenetic relationships and taxonomic position of genus Hyperacrius (Rodentia: Arvicolinae) from Kashmir based on evidences from analysis of mitochondrial genome and study of skull morphology
Source: PeerJ. 2020 Nov 18;8:e10364. doi: 10.7717/peerj.10364 (PMC7680025; doi:10.7717/peerj.10364)

Figure S3. Phylogenetic reconstruction of tribes Arvicolini and Clethrionomyini using alignment with excluded third codon position

The trees were inferred from the concatenated dataset of 13 mitochondrial protein-coding genes. Node labels display BI/ML support, black circles show nodes with 0.95-1.0/95-100 support. Cytochrome *b* sequences are marked with asterisk

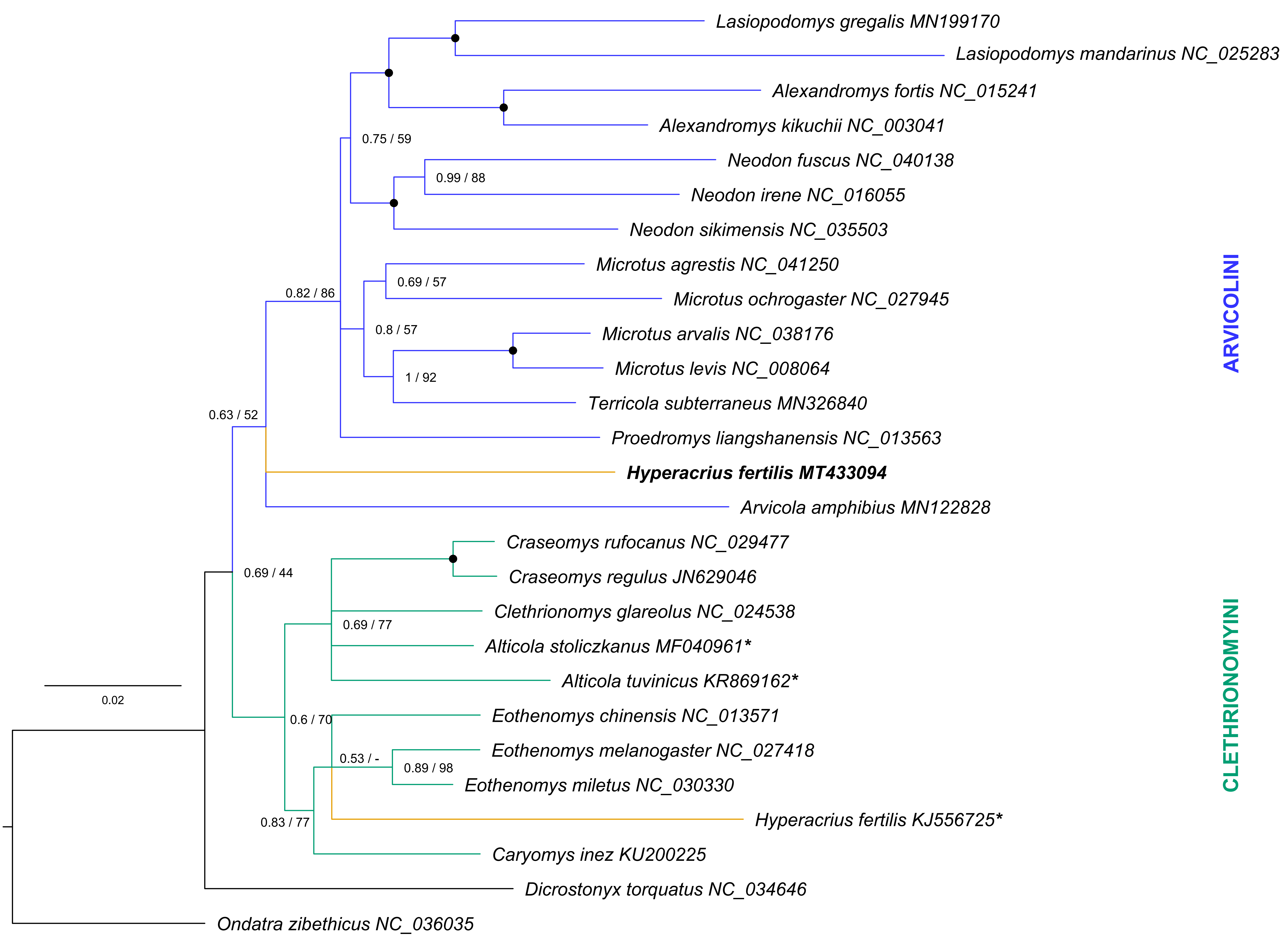

Supplement: Supplemental Information 3 — The trees were inferred from the concatenated dataset of 13 mitochondrial protein-coding genes. Node labels display BI/ML support; black circles show nodes with 0.95-1.0/95-100 support. Cytochrome b sequences are marked with asterisk. [file peerj-08-10364-s003.pdf]
