# Supplementary material for: Phylogenetic relationships and taxonomic position of genus Hyperacrius (Rodentia: Arvicolinae) from Kashmir based on evidences from analysis of mitochondrial genome and study of skull morphology
Source: PeerJ. 2020 Nov 18;8:e10364. doi: 10.7717/peerj.10364 (PMC7680025; doi:10.7717/peerj.10364)
Supplement: Supplemental Information 4 — Bayesian trees inferred from separate PCGs partitioned by codon position. Node labels display BI posterior probabilities (PP). [file peerj-08-10364-s004.pdf]

**Figure S4. Bayesian phylogenetic reconstruction of tribes Arvicolini and Clethrionomyini inferred separately from alignments of 13 mitochondrial protein-coding genes.**
Bayesian trees inferred from separate PCGs partitioned by codon position.
Node labels display BI posterior probabilities (PP).

*ATP6*

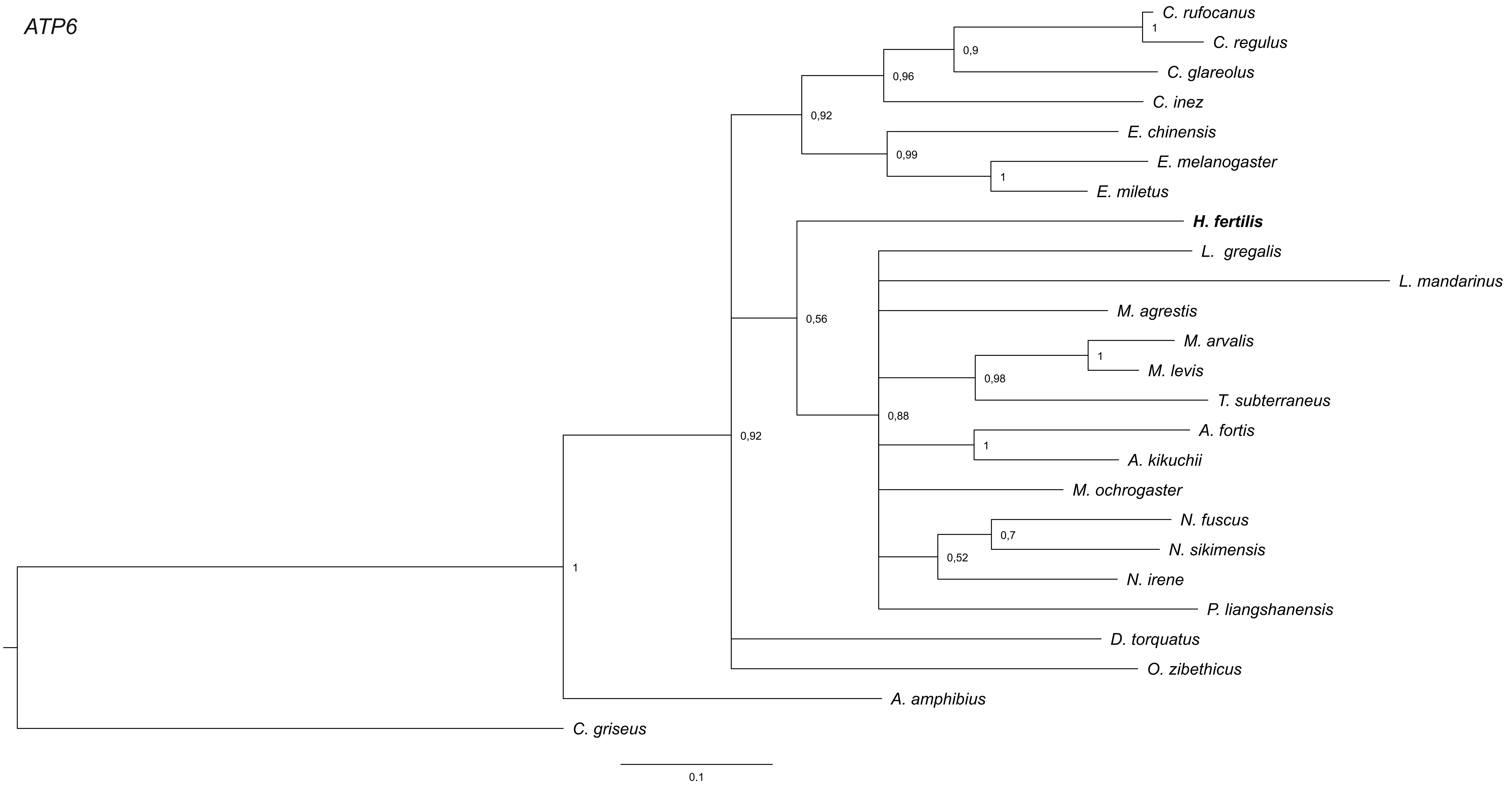

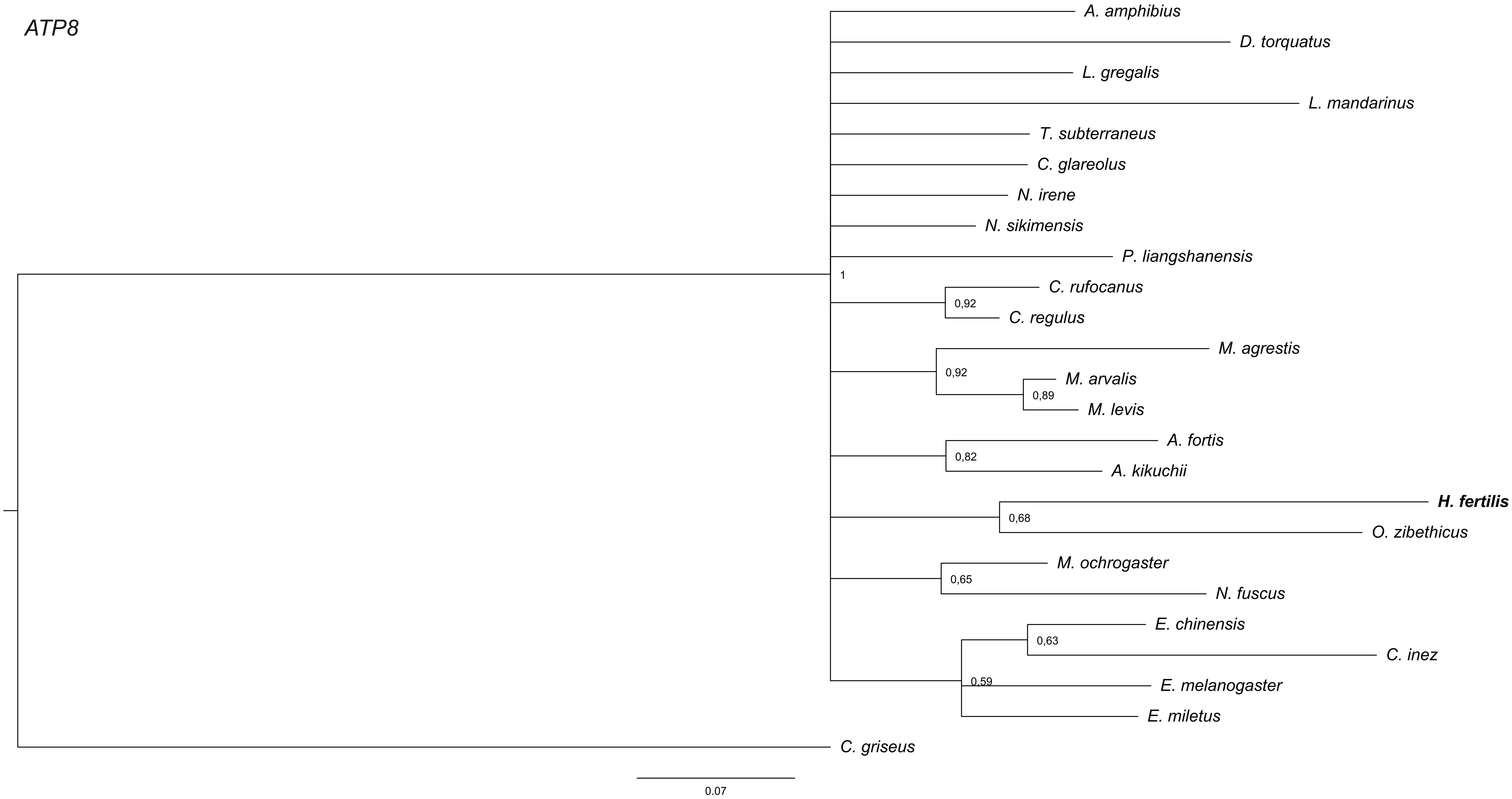

ATP8
A. amphibius
D. torquatus
L. gregalis
L. mandarinus
T. subterraneus
C. glareolus
N. irene
N. sikimensis
P. liangshanensis
1
C. rufocanus
0,92
C. regulus
M. agrestis
0,92
M. arvalis
0,89
M. levis
A. fortis
0,82
A. kikuchii
H. fertilis
0,68
O. zibethicus
M. ochrogaster
0,65
N. fuscus
E. chinensis
0,63
C. inez
0,59
E. melanogaster
E. miletus
C. griseus
0.07

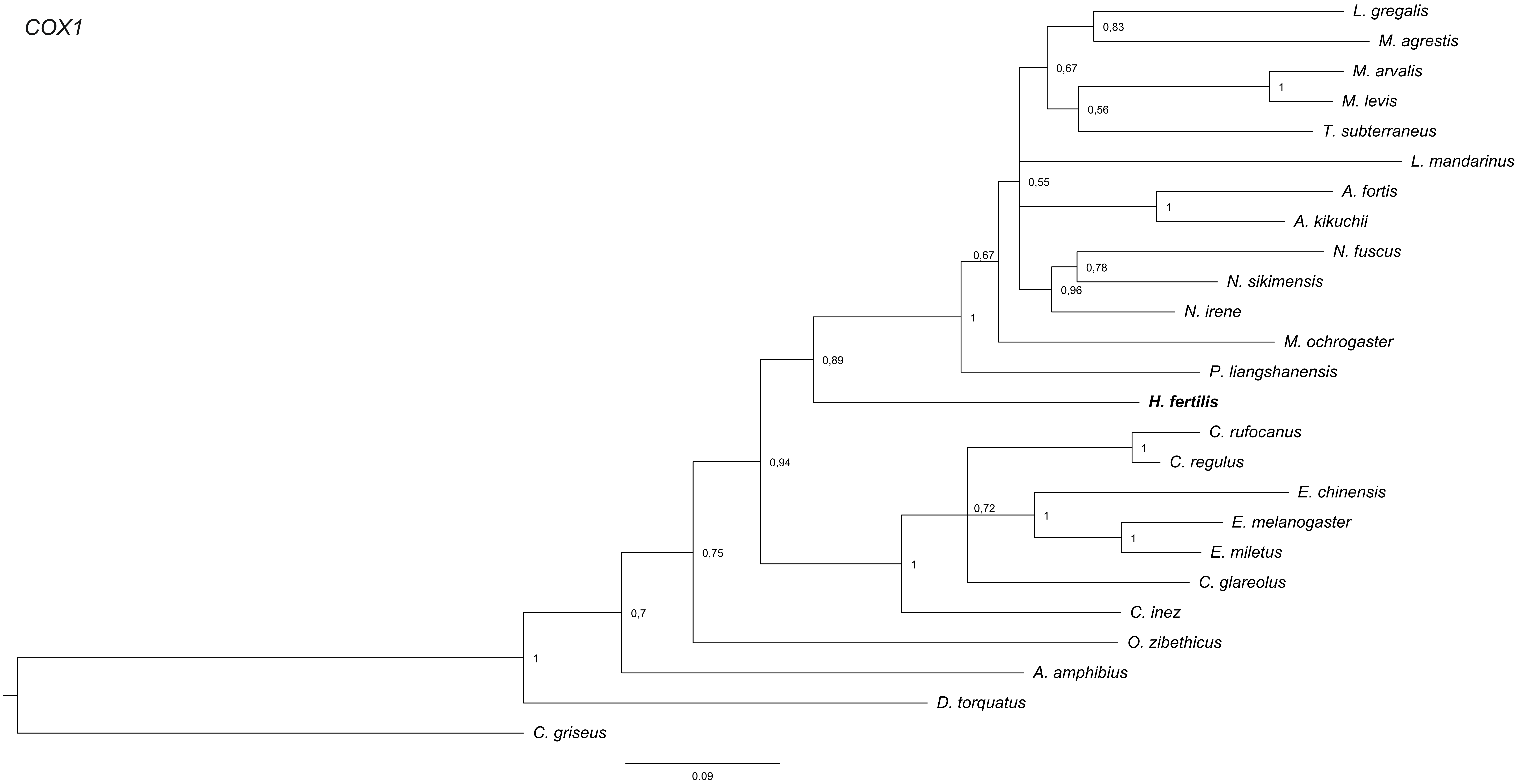
COX1
L. gregalis
M. agrestis
M. arvalis
M. levis
T. subterraneus
L. mandarinus
A. fortis
A. kikuchii
N. fuscus
N. sikimensis
N. irene
M. ochrogaster
P. liangshanensis
H. fertilis
C. rufocanus
C. regulus
E. chinensis
E. melanogaster
E. miletus
C. glareolus
C. inez
O. zibethicus
A. amphibius
D. torquatus
C. griseus
0.09

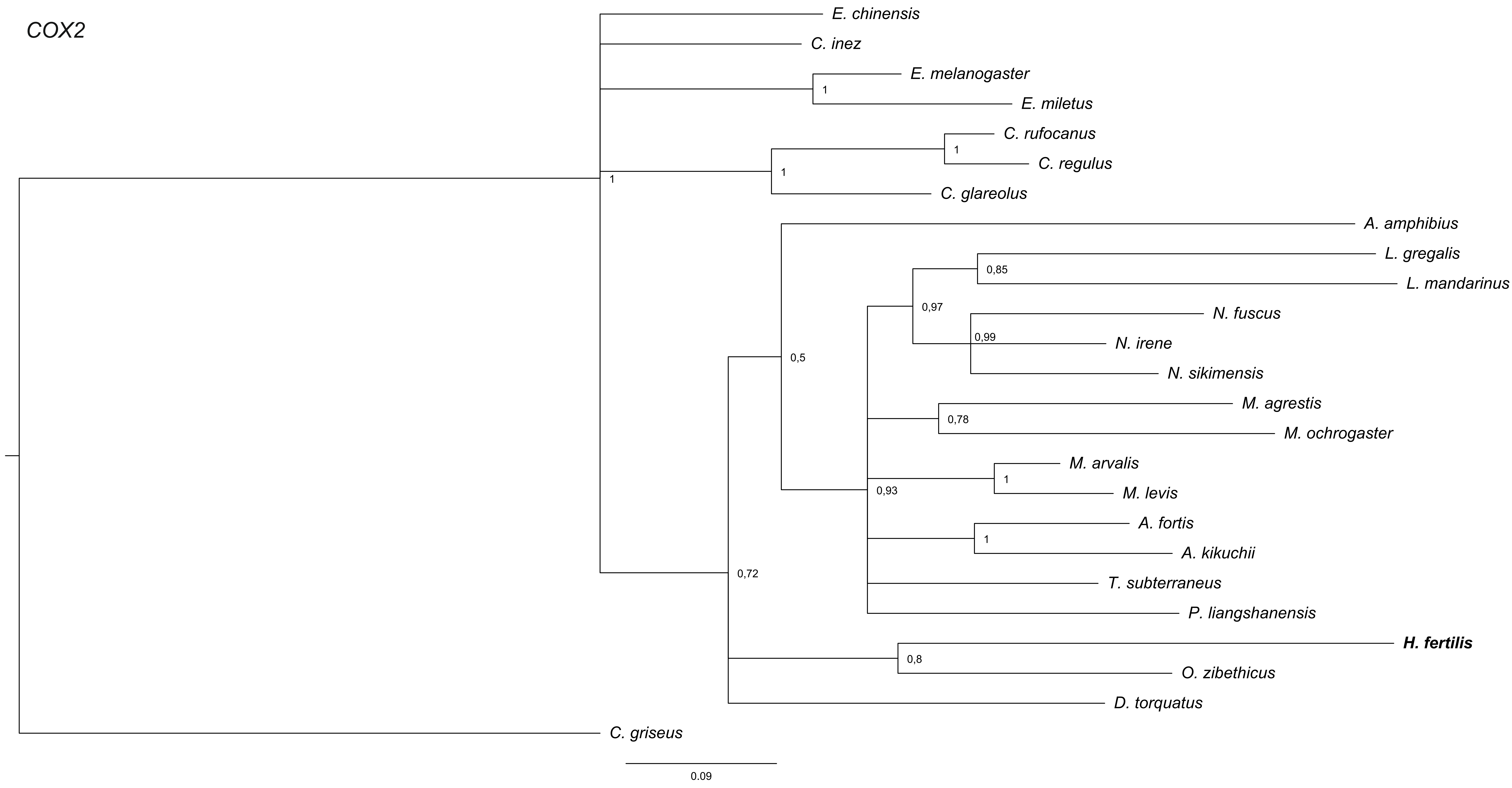
COX2
E. chinensis
C. inez
E. melanogaster
E. miletus
C. rufocanus
C. regulus
C. glareolus
A. amphibius
L. gregalis
L. mandarinus
N. fuscus
N. irene
N. sikimensis
M. agrestis
M. ochrogaster
M. arvalis
M. levis
A. fortis
A. kikuchii
T. subterraneus
P. liangshanensis
H. fertilis
O. zibethicus
D. torquatus
C. griseus
1
1
1
1
0,5
0,85
0,97
0,99
0,78
0,93
1
1
0,72
0,8
0.09

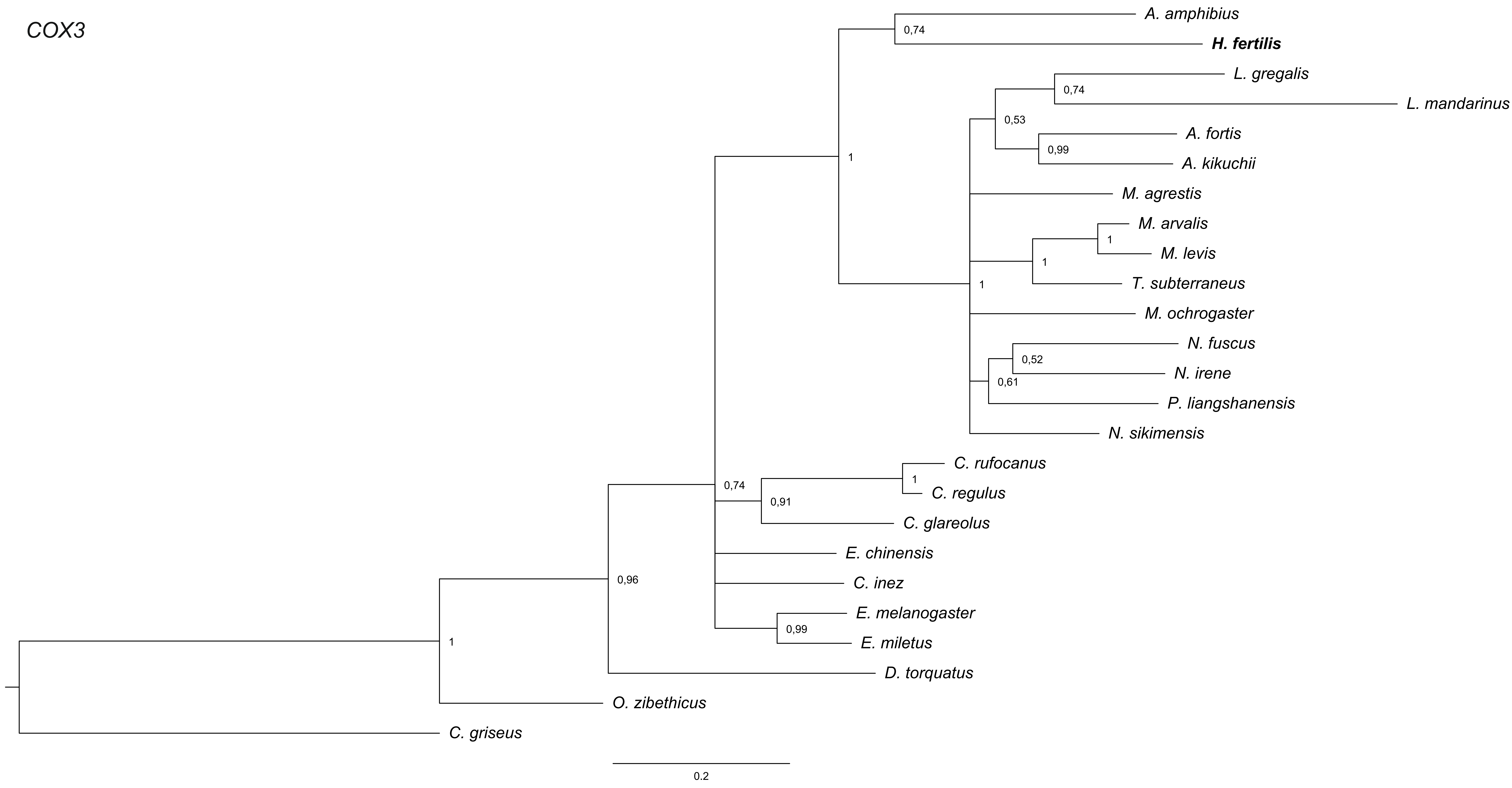
COX3
A. amphibius
0,74
H. fertilis
L. gregalis
0,74
L. mandarinus
0,53
A. fortis
0,99
A. kikuchii
1
M. agrestis
M. arvalis
1
M. levis
1
T. subterraneus
1
M. ochrogaster
N. fuscus
0,52
N. irene
0,61
P. liangshanensis
N. sikimensis
C. rufocanus
1
C. regulus
0,74
0,91
C. glareolus
E. chinensis
C. inez
E. melanogaster
0,99
E. miletus
0,96
D. torquatus
1
O. zibethicus
C. griseus
0.2

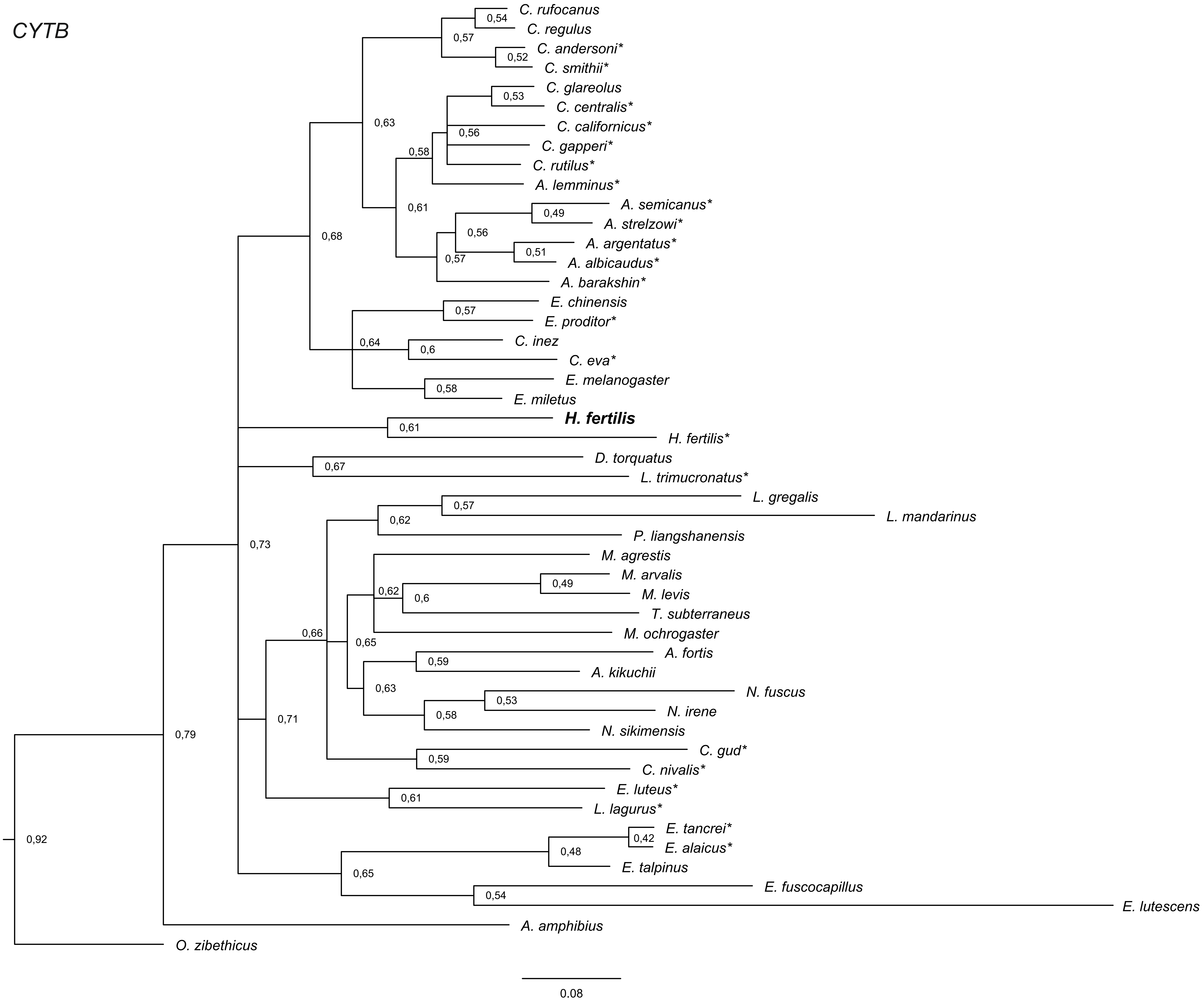


Additional cytochrome *b* sequences downloaded from Genbank are labelled with asterisk.

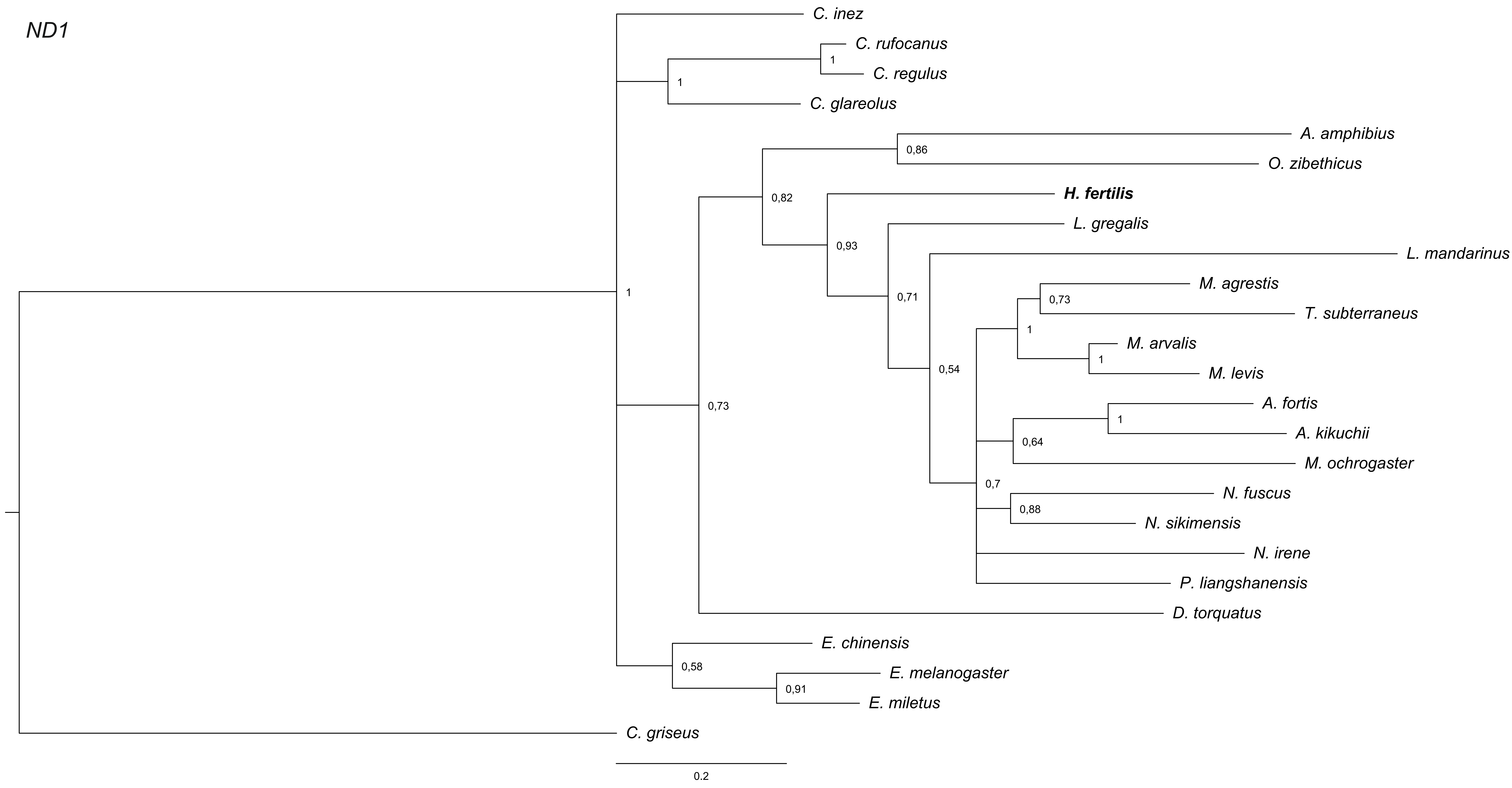
ND1
C. inez
C. rufocanus
C. regulus
C. glareolus
A. amphibius
O. zibethicus
H. fertilis
L. gregalis
L. mandarinus
M. agrestis
T. subterraneus
M. arvalis
M. levis
A. fortis
A. kikuchii
M. ochrogaster
N. fuscus
N. sikimensis
N. irene
P. liangshanensis
D. torquatus
E. chinensis
E. melanogaster
E. miletus
C. griseus
1
1
1
0,86
0,82
0,93
0,71
0,54
0,73
0,73
1
1
1
0,64
0,7
0,88
0,58
0,91
0.2

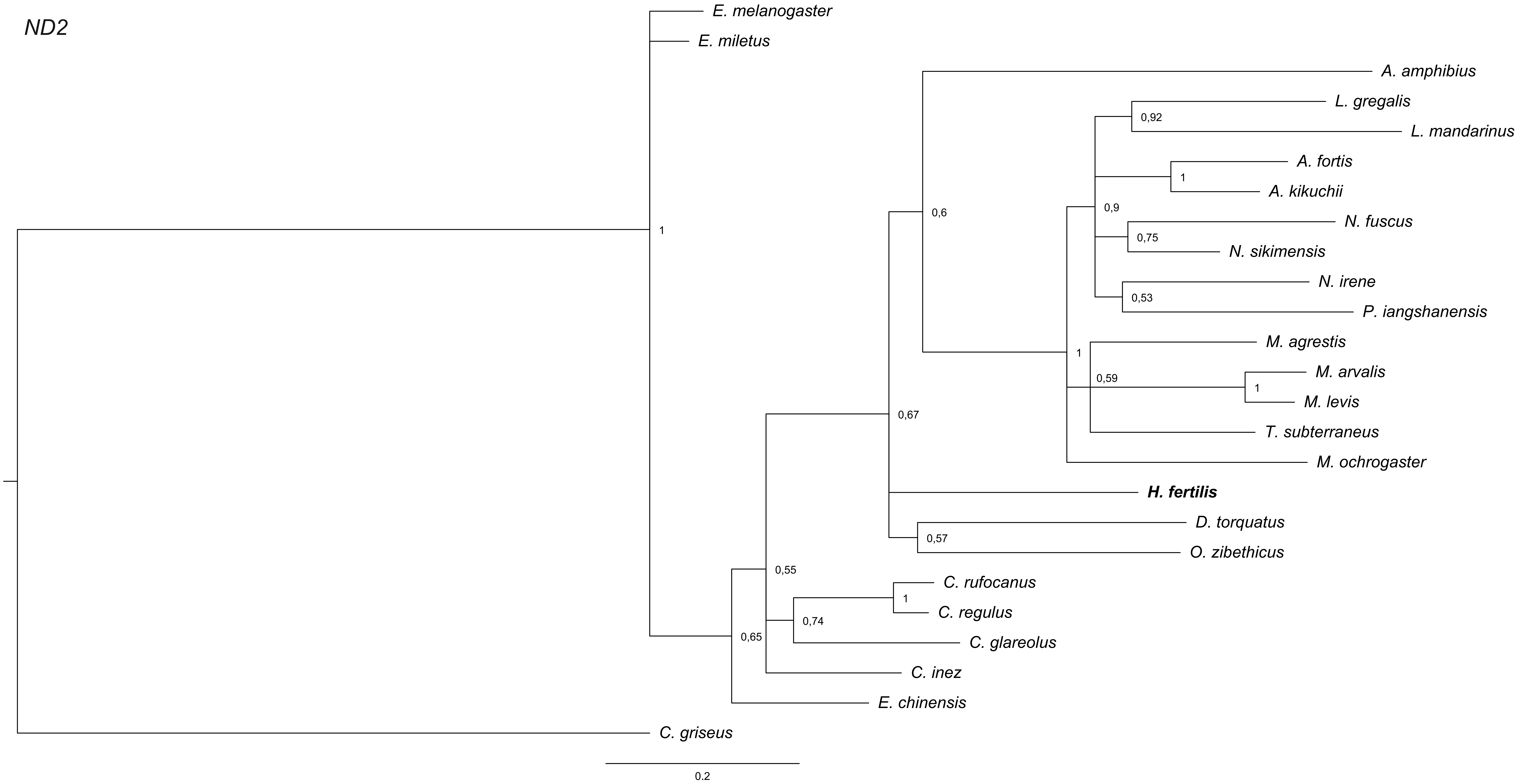
ND2
E. melanogaster
E. miletus
A. amphibius
L. gregalis
0,92
L. mandarinus
A. fortis
1
A. kikuchii
0,9
0,6
N. fuscus
0,75
N. sikimensis
N. irene
0,53
P. iangshanensis
M. agrestis
1
0,59
M. arvalis
1
M. levis
T. subterraneus
M. ochrogaster
0,67
1
H. fertilis
D. torquatus
0,57
O. zibethicus
0,55
C. rufocanus
1
C. regulus
0,74
C. glareolus
0,65
C. inez
E. chinensis
C. griseus
0.2

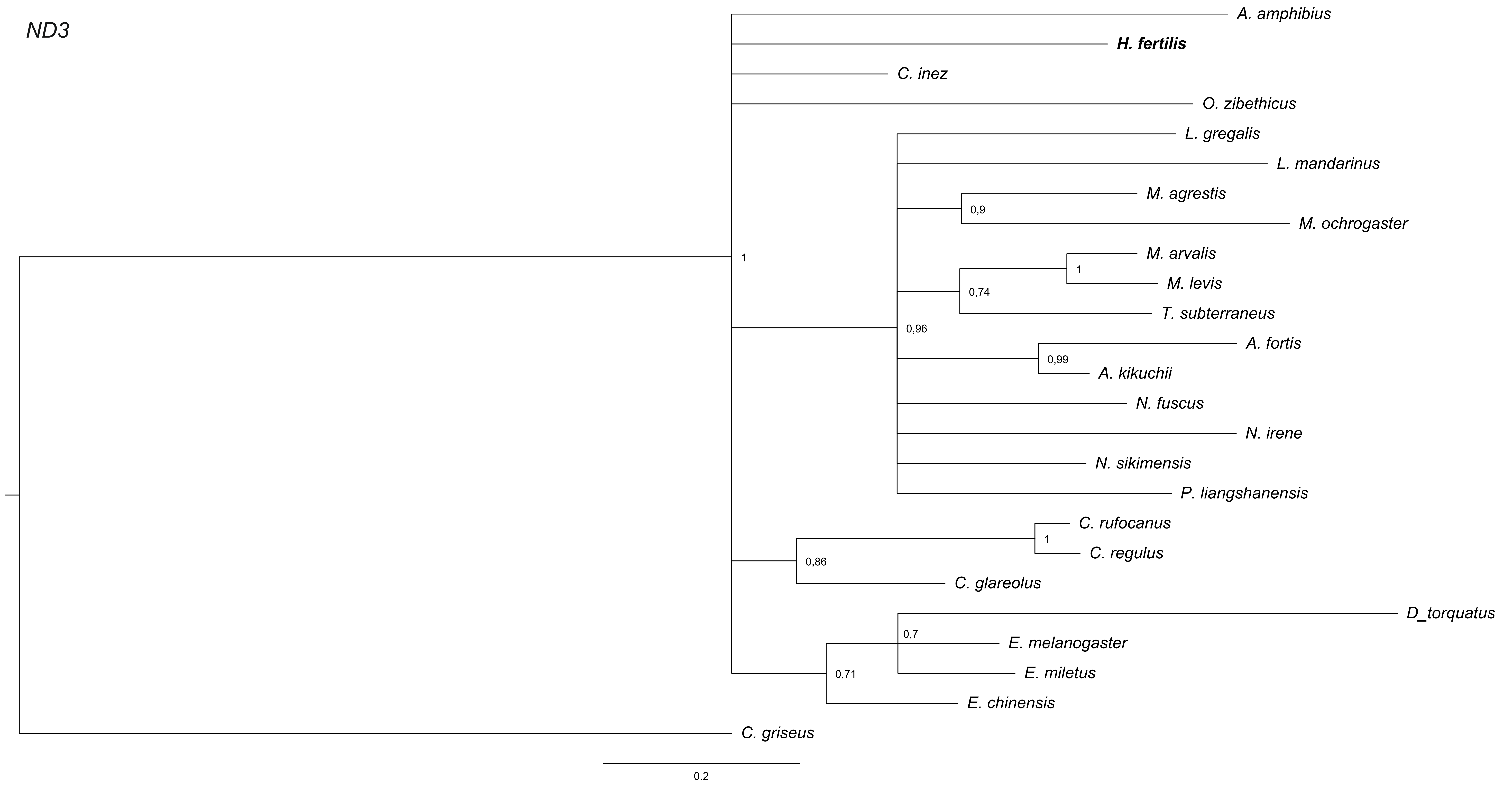
ND3
A. amphibius
H. fertilis
C. inez
O. zibethicus
L. gregalis
L. mandarinus
M. agrestis
0,9
M. ochrogaster
M. arvalis
1
M. levis
0,74
T. subterraneus
1
0,96
A. fortis
0,99
A. kikuchii
N. fuscus
N. irene
N. sikimensis
P. liangshanensis
C. rufocanus
1
C. regulus
0,86
C. glareolus
D_torquatus
0,7
E. melanogaster
E. miletus
0,71
E. chinensis
C. griseus
0.2

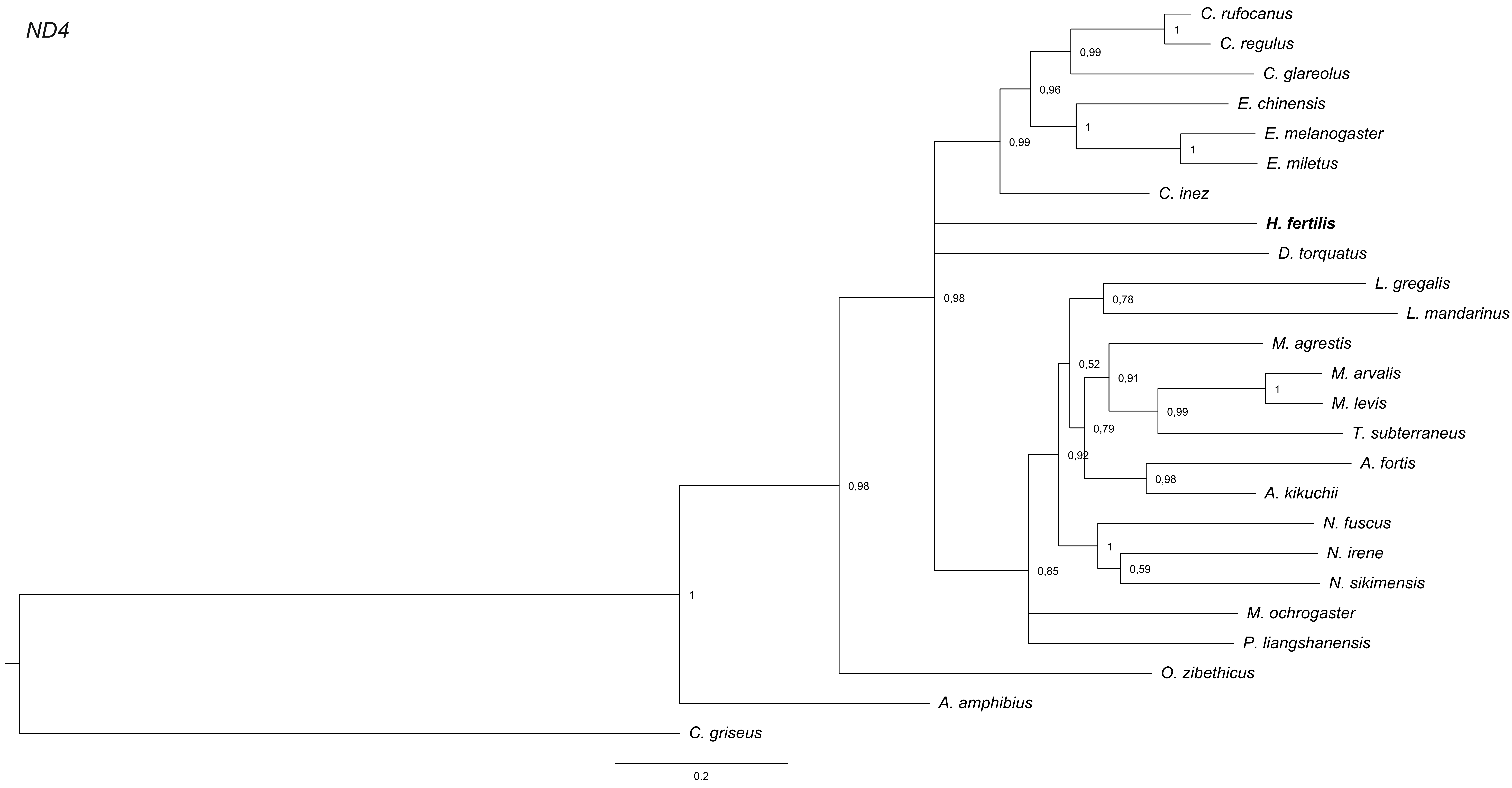
ND4
C. rufocanus
1
C. regulus
0,99
C. glareolus
0,96
E. chinensis
1
E. melanogaster
0,99
1
E. miletus
C. inez
H. fertilis
D. torquatus
L. gregalis
0,98
0,78
L. mandarinus
M. agrestis
0,52
M. arvalis
0,91
1
M. levis
0,99
0,79
T. subterraneus
0,92
A. fortis
0,98
0,98
A. kikuchii
N. fuscus
1
N. irene
0,59
0,85
N. sikimensis
1
M. ochrogaster
P. liangshanensis
O. zibethicus
A. amphibius
C. griseus
0.2

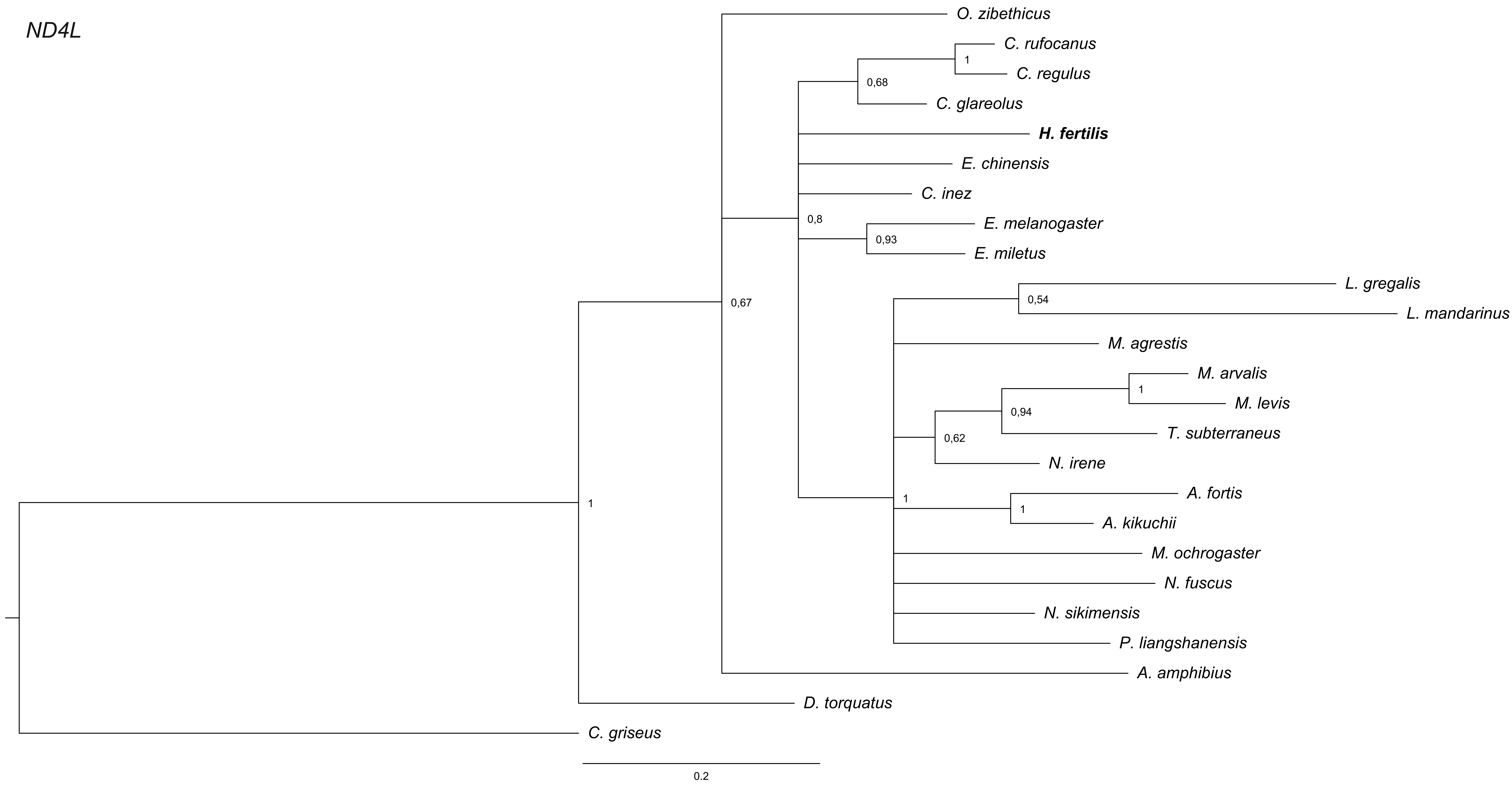

ND4L
O. zibethicus
C. rufocanus
1
C. regulus
0,68
C. glareolus
H. fertilis
E. chinensis
C. inez
0,8
E. melanogaster
0,93
E. miletus
L. gregalis
0,67
0,54
L. mandarinus
M. agrestis
M. arvalis
1
M. levis
0,94
T. subterraneus
0,62
N. irene
1
A. fortis
1
1
A. kikuchii
M. ochrogaster
N. fuscus
N. sikimensis
P. liangshanensis
A. amphibius
D. torquatus
C. griseus
0.2

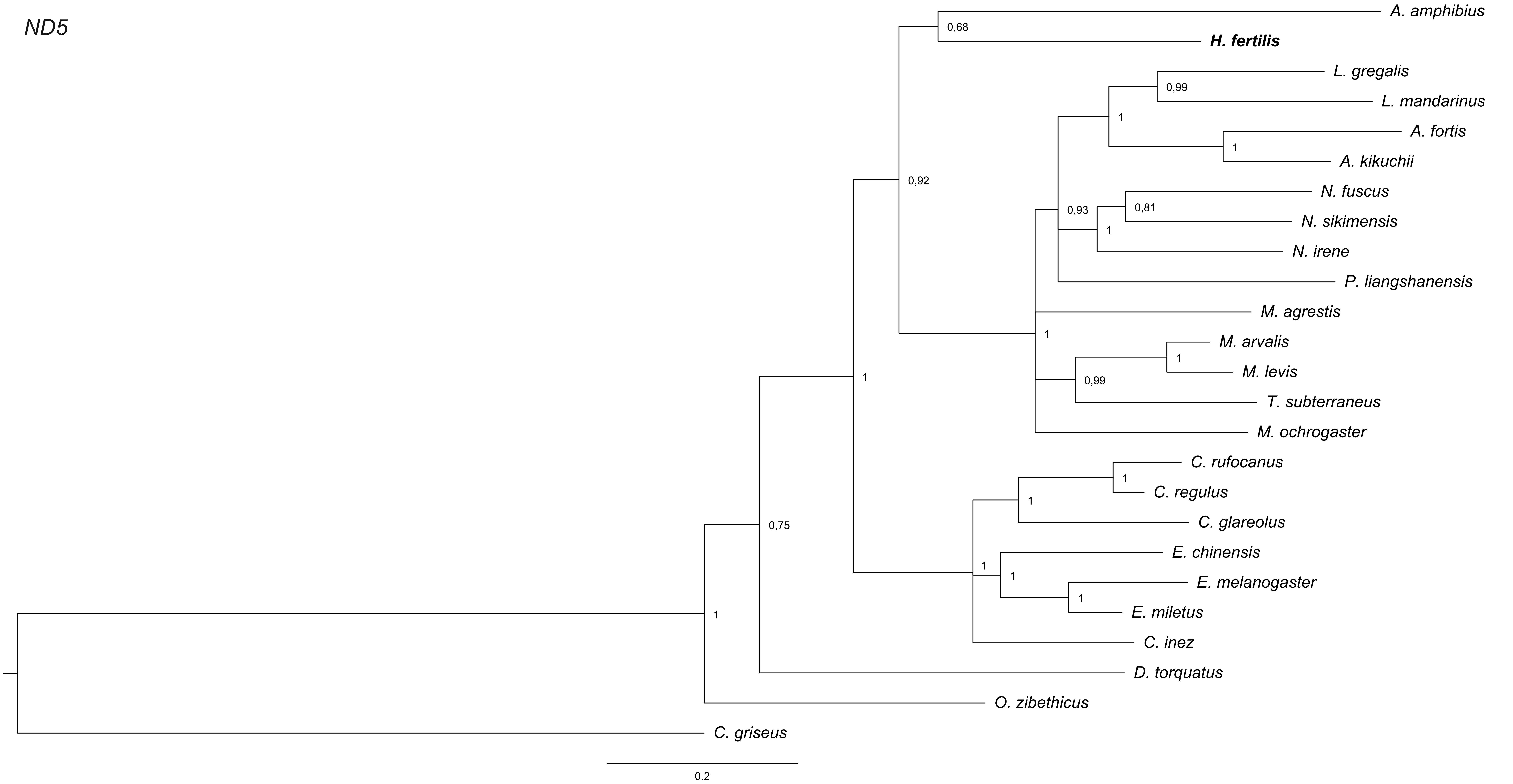
ND5
A. amphibius
H. fertilis
L. gregalis
L. mandarinus
A. fortis
A. kikuchii
N. fuscus
N. sikimensis
N. irene
P. liangshanensis
M. agrestis
M. arvalis
M. levis
T. subterraneus
M. ochrogaster
C. rufocanus
C. regulus
C. glareolus
E. chinensis
E. melanogaster
E. miletus
C. inez
D. torquatus
O. zibethicus
C. griseus
0,68
0,99
1
1
0,92
0,93
0,81
1
1
1
0,99
1
0,75
1
1
1
1
1
1
0.2

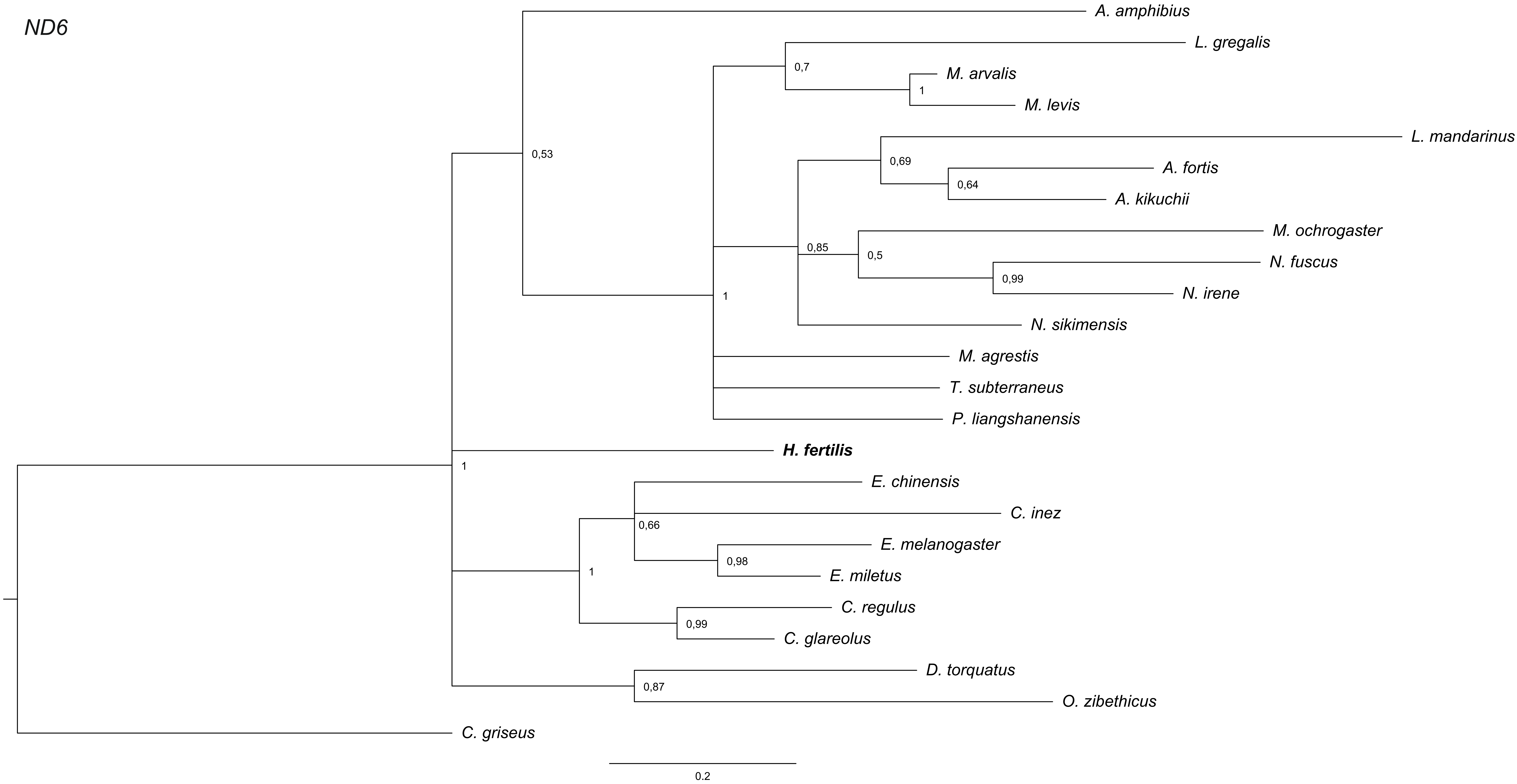
ND6
A. amphibius
L. gregalis
M. arvalis
M. levis
L. mandarinus
A. fortis
A. kikuchii
M. ochrogaster
N. fuscus
N. irene
N. sikimensis
M. agrestis
T. subterraneus
P. liangshanensis
H. fertilis
E. chinensis
C. inez
E. melanogaster
E. miletus
C. regulus
C. glareolus
D. torquatus
O. zibethicus
C. griseus
0,53
0,7
1
0,69
0,64
0,85
0,5
0,99
1
1
0,66
0,98
1
0,99
0,87
0.2
